# Omega-3 Polyunsaturated Fatty Acids Supplements and Cardiovascular Disease Outcome: A Systematic Review and Meta-Analysis on Randomized Controlled Trials

**DOI:** 10.31083/j.rcm2401024

**Published:** 2023-01-12

**Authors:** Xue Qi, Hechen Zhu, Ru Ya, Hao Huang

**Affiliations:** ^1^Department of Critical Rehabilitation, Shanghai Third Rehabilitation Hospital, 200436 Shanghai, China; ^2^Department of Critical Care Medicine, Huashan Hospital, Fudan University, 200040 Shanghai, China

**Keywords:** polyunsaturated fatty acids, cardiovascular disease, randomized controlled trial, meta-analysis

## Abstract

**Background:**

Many meta-analyses and randomized controlled trials (RCTs) on the use of Omega-3 supplements for cardiovascular disease (CVD) have come to different outcomes. Besides, previous meta-analyses have missed some key RCTs on this topic.

**Methods:**

PubMed, EMBASE, Cochrane Library and Web of Science were manually searched for eligible RCTs on Omega-3 polyunsaturated fatty acids (PUFA) use for CVD. Risk estimates of each relevant outcome were calculated as a hazard ratio (HR) with 95% confidence interval (95% CI) using the random-effects model. Subgroup analysis was conducted according to the main characteristics of the population, sensitivity analysis would be performed if there was significant heterogeneity among analyses on relevant outcomes. Statistical heterogeneity was assessed using chi-square tests and quantified using I-square statistics.

**Results:**

Nineteen eligible RCTs incorporating 116,498 populations were included. Omega-3 PUFA supplementation could not significantly improve the outcomes of major adverse cardiovascular events (MACE) (HR: 0.98, 95% CI: 0.91–1.06), myocardial infarction (MI) (HR: 0.86, 95% CI: 0.70–1.05), coronary heart disease (CHD) (HR: 0.90, 95% CI: 0.80–1.01), stroke (HR: 1.00, 95% CI: 0.91–1.10), SCD (sudden cardiac death) (HR: 0.90, 95% CI: 0.80–1.02), all-cause mortality (HR: 0.96, 95% CI: 0.89–1.04), hospitalization (HR: 0.99, 95% CI: 0.81–1.20), hospitalization for all heart disease (HR: 0.91, 95% CI: 0.83–1.00), hospitalization for heart failure (HR: 0.97, 95% CI: 0.91–1.04). Although omega-3 PUFA significantly reduced revascularization (HR: 0.90, 95% CI: 0.81–1.00) and cardiovascular mortality (CV mortality) (HR: 0.91, 95% CI: 0.85–0.97), risk for atrial fibrillation (AF) was also increased (HR: 1.56, 95% CI: 1.27–1.91). Subgroup analysis results kept consistent with the main results.

**Conclusions:**

Omega-3 PUFA supplementation could reduce the risk for CV mortality and revascularization, it also increased the AF incidence. No obvious benefits on other CVD outcomes were identified. Overall, potential CVD benefits and harm for AF should be balanced when using omega-3 PUFA for patients or populations at high risk.

## 1. Introduction

Omega-3 polyunsaturated fatty acids (n-3 PUFA) include α-linolenic acid (ALA), eicosapentaenoic acids (EPA), and docosahexaenoic acids (DHA) [1], among which, ALA is abundant in plant, while EPA and DHA are abundant in marine animals. Fish oil stemming from marine animals is also rich in EPA and DHA. Over the past several decades, numerous population-based epidemiological studies have delineated that higher fish oil intake in the diet can reduce the incidence of cardiovascular events (CV events) [2, 3, 4]. The American Heart Association (AHA) also recommends that patients with coronary heart disease (CHD) take 1 g/d of EPA and DHA supplements as directed by their physicians. Two to four g/d EPA and DHA capsules are recommended for patients with hypertriglyceridemia (HTG) under the guidance of their family doctors for treatment [5]. In this case, n-3 PUFA is desired by patients with cardiovascular disease (CVD) to treat their disease and populations with high-risk factors to prevent CVD. Despite the availability of abundant evidence, outcomes derived from current evidence are still inconsistent [6, 7, 8, 9, 10, 11, 12, 13, 14, 15, 16, 17, 18, 19, 20, 21, 22, 23, 24].

From mechanistic aspects, n-3 PUFA confers protection against a wide range of CVD states including modulating cell membrane function, regulating cardiac rhythm, polishing endothelial function, as well as inhibiting inflammatory, oxidative and thrombotic pathways implicated in atherosclerosis [25, 26, 27]. N-3 PUFA also favors modulating triglyceride-rich lipoprotein metabolism [28]. However, from clinical aspects, there still exists a great deal of controversy on the protective role of n-3 PUFA. Some clinical trials displayed a considerable beneficial profile of n-3 PUFA for reducing all-cause mortality, CV mortality, sudden cardiac death (SCD), CHD, and stroke [10, 29, 30]; while others failed to confirm the protective effect [31]. A recent meta-analysis on this similar topic included 16 randomized controlled trials (RCTs), and revealed that n-3 PUFA could significantly improve CVD outcomes, especially for second prevention on 1 g/d level with taking EPA only [32]. To our best knowledge, meta-analysis fails to report the results on some other key CV outcomes such as the hospitalization rate among participants, or to include several essential trials [13, 14, 21, 23, 24]. Importantly, no previous meta-analysis has ever analyzed the influence of statin and antiplatelet drug use on CVD outcomes with n-3 PUFA intake. Overall, these inconsistent results warrant a better understanding of the effects of n-3 PUFA on comprehensive subtypes of CVD states. Additionally, limitations of previous meta-analyses on a similar topic should be overcome and updated. To this end, the current study aimed to: (1) conduct a systematic review and meta-analysis by incorporating all eligible RCTs; (2) report results on CVD outcomes in a more comprehensive manner; and (3) analyze the influence of statin and antiplatelet drug use on the final results.

## 2. Methods 

This study was conducted based on the Cochrane Handbook and Preferred Reporting Items for Systematic Reviews and Meta-Analyses (PRISMA) guidelines (**Supplementary Table 1**) [33]. The study protocol is consistent with a previous meta-analysis [32] and has been registered on the INPLASY website (https://inplasy.com/) with a reference ID: INPLASY2022110027 (doi: 10.37766/inplasy2022.11.0027) (**Supplementary Table 2**).

### 2.1 Search Strategy

We reviewed databases of Pubmed, EMBASE, Cochrane Library and Web of Science for eligible studies from the inception to Aug-15-2022. The combined search strategy of relevant keywords and Medical Subject Headings (MeSH) terms used in current study are: “Omega-3 fatty acids”, “docosahexaenoic acid”, “DHA”, “Eicosapentaenoic acid”, “EPA”, “cardiovascular disease”, “cardiovascular events”, “coronary heart disease”, “myocardial infarction”, “stroke” and “randomized controlled trial”. A detailed search strategy has been given in Table 1. No special restrictions were applied to language. Reference lists of the retrieved literature were also searched manually.

**Table 1.** **Literature search strategy for relevant databases**.

1 Omega-3 fatty acids	(“Omega-3 fatty acids” [Mesh] OR “Omega-3 Fatty Acid” OR “Omega 3 Fatty Acid” OR “n-3 Oil” OR “n-3 Fatty Acids” OR “Omega 3 Fatty Acids” OR “n-3 PUFA” OR “n3 Fatty Acid” OR “n3 Polyunsaturated Fatty Acid” OR “n-3 Oils” OR “N-3 Fatty Acid” OR “Fatty Acid, N-3” OR “n-3 Polyunsaturated Fatty Acid” OR “n 3 Polyunsaturated Fatty Acid” OR “Oil, n-3” OR “Omega 3 Fatty Acids” OR “PUFA, n3”)
2 Cardiovascular disease	(“Cardiovascular disease” OR “Disease, cardiovascular” OR “Diseases, cardiovascular” OR “Coronary disease” OR “Coronary heart disease” OR “Disease coronary heart” OR “Myocardial infarction” OR “Infarct, myocardial” OR “Heart Attack” OR “Heart attacks” OR “Stroke” OR “Cerebrovascular accident” OR “Brain vascular accident” OR “Cerebral stroke” OR “Acute cerebrovascular accident” OR “Apoplexy”)
3 Randomized controlled trials	(Randomized controlled trials[pt] OR Randomized controlled trial[pt] OR Clinical Trials, Randomized[pt] OR Trials, Randomized Clinical[pt] OR Controlled Clinical Trials, Randomized[pt])
4	(animals[mh] NOT humans[mh])
5	1 AND 2 AND 3
6	5 NOT 4

### 2.2 Selection Criteria

All searched articles went through a two-step review process. They were initially screened for titles and abstracts. Then, the full texts of possibly eligible studies were reviewed by two independent authors (Xue Qi and Hao Huang). Any disagreements were resolved by a discussion in a group panel with another author (Ru Ya), who is familiar with cardiology and evidence-based medicine.

The eligible criteria following the PICOS principles were listed as:

**Populations**: Adult populations (≥18 yr) with CVD or high-risk factors (e.g., smoking, obesity, lack of physical activity, etc.) for CVD; and no restrictions on their gender, race, nationality and CV-related comorbidities (e.g., diabetes, hypertension, kidney circulation dysfunction).

**Intervention/comparison**: Omega-3 PUFA from dietary supplements, capsules or drug prescriptions was used. Considering the difficulty in quantifying n-3 PUFA intake from marine fish food sources, Omega-3 PUFA directly derived from these resources was considered not eligible.

**Outcomes**: At least one of the following outcomes was reported with available data for calculating: major adverse cardiovascular events (MACE), myocardial infarction (MI), CHD, revascularization, stroke, sudden cardiac death (SCD), CV mortality, all-cause mortality, hospitalization, hospitalization for all heart disease, hospitalization for heart failure, and atrial fibrillation (AF).

**Study design**: Randomized controlled trial (RCT).

The same trial with a longer follow-up period could be included to avoid duplication. Eligible RCTs should have a registered protocol, and provide ethics approval and consent of individuals. Observational studies, reviews, case reports, conference abstracts and experimental studies were excluded. Studies without essential data were also excluded.

### 2.3 Data Extraction and Outcome of Interest

Data extraction was performed by two independent authors (Xue Qi and Ru Ya) following a pre-ruled protocol from included studies. The extracted information included characteristics of eligible studies (year of publication, first author name, performed country, trial name, follow-up period, etc.), characteristics of the populations (gender (proportion of male), mean age (SD) and sample size (in experimental and control groups), etc.), and the characteristics of the program (interventions in two groups (n-3 PUFA or placebo or other dietary supplements), dose of n-3 PUFA (1–4 g/d), type of n-3 PUFA (EPA + DHA and EPA alone), prevention type (secondary and mixed), registration number, etc.). The risk estimates of hazard ratio (HR) relative risk (RR) and odds ratio (OR) would be evaluated in fully adjusted models if available. If not, the unadjusted models would be evaluated, and special descriptions would be given. Intentional-to-treat (ITT) principles would be applied if available. The authors would contact the primary authors for some missing data to facilitate the current analysis, and the current study would still have been taken without these data if no response was received.

Herein, outcomes including MACE, MI, CHD, revascularization, stroke, SCD, CV mortality, all-cause mortality, hospitalization, hospitalization for all heart disease, and hospitalization for heart failure and AF were analyzed. Details about the definitions on these outcomes were summarized in **Supplementary Table 3**. Briefly, MACE indicated a composite of MI, stroke, cardiac death or any revascularization; MI included fatal and no-fatal MI; stroke included fatal and no-fatal stroke; and AF meant new AF events.

### 2.4 Quality Assessment

For evaluating the quality of included studies, we applied the Cochrane Risk of Bias Tool, which has been widely used for assessing the methodological quality of RCTs in meta-analyses [34]. Seven specific bars in the Cochrane Risk of Bias Tool were objectively evaluated by two independent authors (Xue Qi and Ru Ya) including the generation of randomized sequences, concealment of allocation protocols, blinding of study participants and related persons, blinding of outcome evaluators, incomplete data on study results, selective reporting of results and other sources of bias. If each bar from the Cochrane Risk of Bias Tool was not available or wrongly conducted, assessment on the bar would be high risk.

### 2.5 Statistical Analysis

Fully adjusted HR and the corresponding 95% confidence intervals (95% CIs) for the outcomes of interests obtained from Cox-Hazard regression analysis were mainly estimated with DerSimonian-Laird (D-L) random effects model because the assumptions might be attributed to the presence of whining-study and between-study heterogeneity. The adjusted/unadjusted RR and OR in primarily included studies were approximately considered as HR. HRs and standard errors (SEs) originating from the correspondence 95% CIs were logarithmically transformed to stabilized variance, and the distribution then was normalized. Between-study heterogeneity was determined with the Cochran Q chi-square test and the *$I^{2}$*. An *$I^{2}$* >50% or a *p* value for the Q test <0.1 was regarded as equal to significant heterogeneity [35].

Sensitivity analysis would be performed by removing one study each turn to reduce and elaborate the causes of the heterogeneity in the case of significant heterogeneity. Post-subgroup analyses were also conducted to ascertain the influence of other risk factors on the outcome results on MACE, CV mortality and all-cause mortality, since there were abundantly included studies on those outcomes. According to the main characteristics of the populations and trial, the subgroups were identified as follows: the proportion of statin use populations (<50% vs. ≥50%) in each trial, proportion of antiplatelet drug use populations (<50% vs. ≥50%) in each trial, n-3 PUFA formulations (EPA + DHA vs. EPA) in each trial, actual amount of n-3 PUFA intake (<2 g/d vs. ≥2 g/d) in each trial and prevention type (primary prevention vs. secondary prevention vs. mixed prevention) in each trial. The analyses results of the subgroup were visualized by forest plots.

Publication bias was estimated using Begg’s correlation test and Egger’s linear regression test at *p *< 0.10 indicating significant publication bias [36]. All analyses were performed using Stata software version 12.0 (https://www.stata.com/); two-sided *p *< 0.05 was considered statistically significant. When 95% CIs of HR were on 1.00, the *p* value for HR would be checked, with *p *< 0.05 indicating statistical significance.

## 3. Results

### 3.1 Study Selection and Characteristics of the Included Studies

**Fig. 1.**
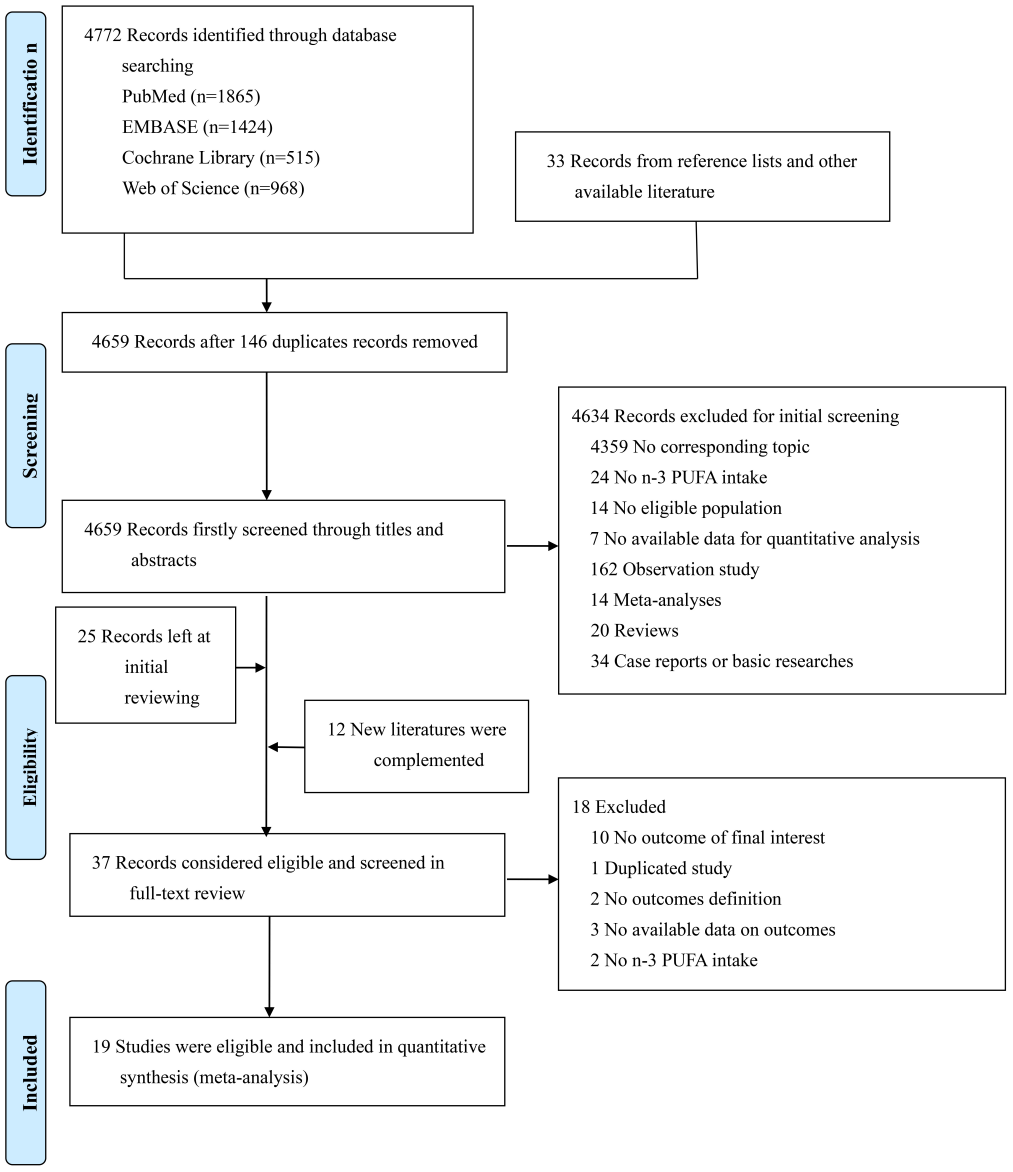
**The flow chart for study screening and selection**.

Of 4772 studies searched from databases, 1865 came from PubMed, 1424 from EMBASE, 515 from Cochrane Library, and 968 from Web of Science. Additionally, 33 were further achieved from other literature available. Then, 4634 records were excluded after initial screening, 12 new records were complemented by reviewing reference list when making the initial screening, and 18 were excluded after full-text consideration due to no outcome of interest or definition, duplicated study, no useful data or no n-3 PUFA intake. Finally, a total of 19 studies were eligible for systematic review and meta-analysis, and the selection process and exclusion reasons could be found in Fig. 1.

Totally, 19 RCTs incorporating 116,498 populations were considered eligible and included in current systematic review and meta-analysis (Table 2, Ref. [6, 7, 8, 9, 10, 11, 12, 13, 14, 15, 16, 17, 18, 19, 20, 21, 22, 23, 24]). Ten studies [6, 7, 9, 11, 12, 13, 15, 18, 21, 24] were conducted in Europe, 1 [19] in the USA, 2 [10, 20] in Asia, and the other 6 [8, 14, 16, 22, 23, 24] were performed on multicenter individuals. Only one study [12] was conducted on all male populations. Among all 19 clinical trials, proportion of statin use ≥50% among individuals was observed in 10 studies [10, 13, 14, 15, 16, 20, 21, 22, 23, 24], and <50% in 8 studies [6, 7, 8, 9, 11, 12, 18, 19]; proportion of antiplatelet drug use ≥50% among individuals was observed in 11 studies [6, 9, 11, 13, 14, 15, 16, 17, 20, 23, 24], and <50% in 4 studies [7, 10, 18, 21]. Almost all included studies [6, 7, 8, 9, 11, 12, 13, 15, 16, 17, 18, 19, 21, 23, 24] used combined EPA + DHA for n-3 PUFA supplementation, three [10, 20, 22] used only EPA for n-3 PUFA intake, and one [14] used EPA + DHA + ALA for n-3 PUFA intake. Six studies [7, 8, 12, 22, 23, 24] provided ≥2 g/d n-3 PUFA for the included populations, and 9 [6, 9, 10, 11, 15, 16, 17, 18, 21] provided <2 g/d n-3 PUFA for the included populations. As for the control group prescription, 16 studies [7, 8, 9, 11, 12, 13, 15, 16, 17, 18, 19, 20, 21, 22, 23, 24] used placebo only, one [6] did not provide any treatment for participants in the control group, one [10] provided standard of care, and one [14] provided placebo and ALA. Eight studies [6, 7, 9, 13, 14, 15, 20, 24] were designed for the secondary prevention of CVD, 10 [8, 10, 11, 12, 16, 17, 18, 19, 22, 23] for mixed (primary and secondary) prevention for CVD, and only one [21] for the primary prevention for CVD. The mean follow-up period was 3.4 yr. Besides, three studies [15, 18, 19] reported unadjusted results, while the rest reported fully adjusted results based on adjustment on variables such as age, serum glucose, body mass index, systolic blood pressure, and smoking status at baseline.

**Table 2.** **Characteristics of included studies**.

Study	Trial name	Study population	Total population (Experimental/Control group)	Male (%)	Mean age (yr)	Statin use (%)	Antiplatelet drugs use (%)	n-3 FA formulations	Actual amount of free fatty acid	Control	Median follow-up (yr)	Randomization time	Prevention	Outcomes
Marchioli *et al*. (1999); Italy [6]	GISSI-P	participants with MI	11,324 (5666/5658)	85	59.4	4.7	91.7	460 mg EPA + 380 mg DHA (capsule)	1 g/d PUFA	no treatment	3.5	1993–1995	Secondary prevention	⑤⑥⑦⑧
Nilsen *et al*. (2001); Norway [7]	NA	participants with MI	300 (150/150)	79	64	7.2	20.7	850–882 mg EPA + DHA (capsule)	4 g/d PUFA	placebo (corn oil)	2	ended at 1997	Secondary prevention	①②③④⑥⑦⑧
Brouwer *et al*. (2006); Multicenter [8]	SOFA (NCT00110838)	participants with ICDs and malignant VT or VF	546 (273/273)	85	61.5	45.5	NA	464 mg EPA + 335 mg DHA (capsule)	2 g/d PUFA	placebo (sunfloweroil)	1	2001–2004	Mix prevention	②⑧
Svensson *et al*. (2006); Denmark [9]	OPACH	participants with CVD and with chronic HD	206 (103/103)	65	67	19.5	71.4	EPA + DHA (capsule)	1.7 g/d PUFA	placebo (olive oil)	2	2002–2003	Secondary prevention	①②③⑤⑧
Yokoyama *et al*. (2007); Japan [10]	JELIS (NCT00231738)	participants with 6.5 mmol/L total cholesterol (4.4 mmol/L LDL)	18,645 (9326/9319)	32	61	100	13.9	1800 mg EPA (capsule)	1.8 g/d PUFA	standard of care	5	1996–1999	Mix prevention	②③⑤⑥⑦⑧
Tavazzi *et al*. (2008); Italy [11]	GISSI-HF (NCT00336336)	participants with HF	6975 (3494/3481)	78	67	22.3	58.4	850–882 mg EPA + DHA (capsule)	1 g/d PUFA	placebo	3.9	2002–2005	Mix prevention	②⑤⑥⑦⑨⑩⑪
Einvik *et al*. (2010); Norway [12]	DOIT	participants at high risk for atherosclerosis	563 (282/281)	100	70.1	19	NA	EPA + DHA (capsule)	2.4 g/d PUFA	placebo (corn oil)	2.4	1997–1998	Mix prevention	①⑧
Galan *et al*. (2010); France [13]	SU.FOL.OM3 (ISRCTN41926726)	participants with CVD	2501 (1253/1248)	79.2	60.7	86.4	94	600 mg EPA + DHA (capsule)	NA	placebo	4.7	2003–2007	Secondary prevention	①③④⑤⑧
Kromhout *et al*. (2010); Multicenter [14]	Alpha Omega (NCT00127452)	participants with MI	4837 (2404/2433)	78.2	69	86	97.5	226 mg EPA + 150 mg DHA+ 1.9 g ALA/d (margarine)	NA	placebo + ALA	3.4	2002–2006	Secondary prevention	①⑦⑧
Rauch *et al*. (2010); Germany [15]	OMEGA (NCT00251134)	participants with MI	3818 (1925/1893)	74	64	94.2	81.5	425 mg PA + 345 mg DHA (capsule)	1 g/d PUFA	placebo (olive oil)	1	2003–2007	Secondary prevention	①④⑥⑦⑧
Bosch *et al*. (2012); Multicenter [16]	ORIGIN (NCT00069784)	participants with or at high risk for CVD and diabetes	12,536 (6281/6255)	65	63.5	53	69.1	425 mg EPA + 345 mg DHA (capsule)	1 g/d PUFA	placebo (olive oil)	6.2	2003–2005	Mix prevention	①②④⑤⑦⑧⑩⑪
Macchia *et al*. (2013); Multicenter [17]	FORWARD (NCT00597220)	participants with symptomatic AF	586 (289/297)	55	66.1	NA	50.9	850–882 mg EPA + DHA	1 g/d PUFA	placebo (olive oil)	1	2008–2011	Mix prevention	①⑧⑨⑪⑫
Roncaglioni *et al*. (2013); Italy [18]	R&P study (NCT00317707)	participants with or at high risk for CVD without MI	12,513 (6244/6269)	62	64	42.5	41.3	425 mg PA + 345 mg DHA (capsule)	1 g/d PUFA	placebo (olive oil)	5	2004–2007	Mix prevention	①③⑥⑦⑩
Bonds *et al*. (2014); USA [19]	AREDS2 (AREDS2)	participants with ophthalmological disease, with or without CVD	3159 (2074/1012)	43	74	44	NA	650 mg PA + 350 mg DHA	NA	placebo	4.8	2006–2008	Mix prevention	⑦
Nosaka *et al*. (2017); Japan [20]	NA (UMIN000016723)	participants with ACS	238 (119/119)	76	70.5	100	100	1800 mg EPA	NA	placebo	1.8	2010–2014	Secondary prevention	④⑦⑩⑪
Bowman *et al*. (2018); UK [21]	ASCEND (NCT00135226)	participants with diabetes, without CVD	15,480 (7740/7740)	63.3	62.6	75.3	35.6	425 mg EPA + 345 mg DHA (capsule)	1 g/d PUFA	placebo (olive oil)	7.4	2005–2011	Primary prevention	①④⑤⑦⑧
Bhatt *et al*. (2019); Multicenter [22]	REDUCE-IT (NCT01492361)	participants with or at high risk for CVD	8179 (4089/4090)	71	64	100	NA	3500 mg EPA (IPE)	4 g/d PUFA	placebo (mineral oil)	4.9	2011–2016	Mix prevention	①②④⑤⑦⑧⑩
Nicholls *et al*. (2020); Multicenters [23]	STRENGTH (NCT02104817)	participants with or at high risk for CVD	13,078 (6539/6539)	62.5	65	100	71.3	300 mg EPA + DHA (capsule)	4 g/d PUFA	placebo (corn oil)	3.2	2014–2017	Mix prevention	①③④⑦⑧⑪⑫
Kalstad *et al*. (2021); Norway [24]	OMEMI (NCT01841944)	participants with ACS	1014 (505/509)	74	71	96.4	100	930 mg EPA + 660 mg DHA (capsule)	3.8 g/d PUFA	placebo	2	2012–2018	Secondary prevention	①②④⑤⑧⑪⑫

Abbreviations: FA, fatty acids; MI, myocardial infraction; ICD, implantable cardioverter-defibrillators; VT, ventricular tachycardia; VF, ventricular fibrillation; CVD, cardiovascular disease; HD, chronic hemodialysis; LDL, low density lipoprotein; HF, heart failure; AF, atrial fibrillation; ACS, acute coronary syndrome; EPA, eicosapentaenoic acid; DHA, docosahexaenoic acid; ALA, alpha-linolenic acid; PUFA, polyunsaturated fatty acids. Outcomes: ①MACE, ②MI, ③CHD, ④Revascularization, ⑤Stroke, ⑥Sudden cardiac death, ⑦CV mortality, ⑧All-cause mortality, ⑨Hospitalization, ⑩Hospitalization for all heart disease, ⑪Hospitalization for heart failure, ⑫AF.

In terms of the study methodological quality, both Nilsen *et al*. [7] and Einvik *et al*. [12] failed to provide detailed descriptions on the blinding methods. Nilsen *et al*. [7] did not provide evidence to support the process of randomization and allocation concealment; Einvik *et al*. [12] did not provide any information about allocation concealment as well. Bowman *et al*. [21] carried out an open-label study without taking blinding for participants and outcome assessments. Brouwer *et al*. [8], Kromhout *et al*. [14] and Bonds *et al*. [19] provided incomplete outcome data. Other sources of bias remained unclear in Marchioli *et al*. [6], Svensson *et al*. [9], Einvik *et al*. [12], Macchia *et al*. [17], Bonds *et al*. [19] and Nosaka *et al*. [20](**Supplementary Table 4**).

### 3.2 MACE

**Fig. 2.**
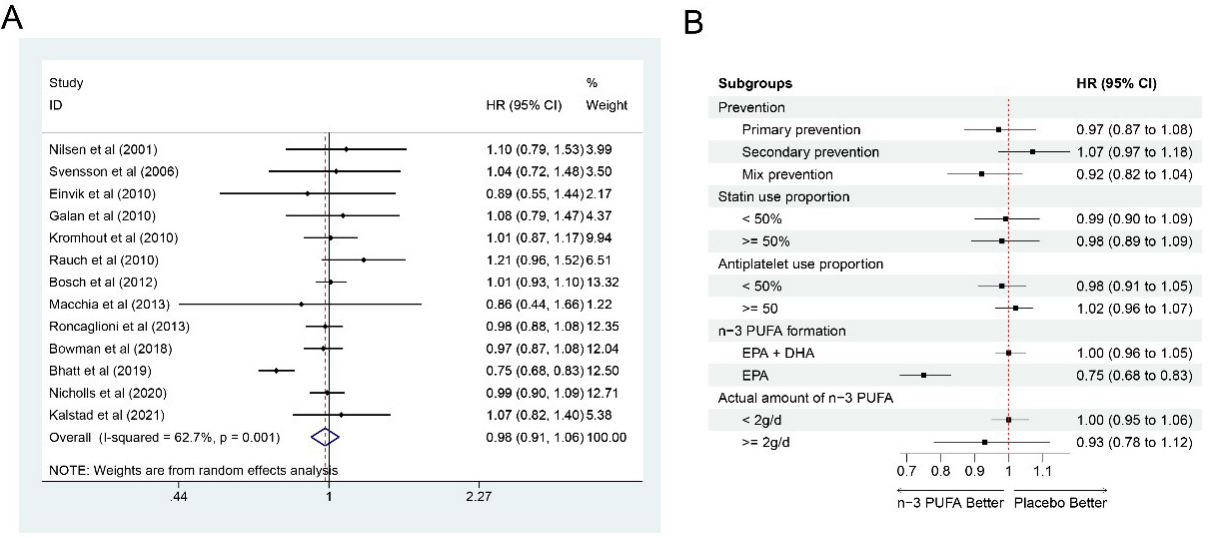
**Forrest plots and subgroup analyses for MACE**. (A) Forest plot of main result on MACE. Each bar and the middle diamond represented HR and 95% CI for each included study with the detailed value marked on right. The bottom diamond represented the synthesized result, if the whole diamond located on the left of vertical full line (on 1.00), the risk of MACE was significantly reduced, if the whole diamond located on the right of vertical full line, the risk of MACE was significantly improved, otherwise there was no statistically significant association. Vertical dashed line in red represented location of synthesized HR from which we could presume the trend of the association. (B) Forest plot of subgroup analysis. Each outcome included a couple of subgroups, the reference list of studies in each subgroup has been marked in the forest plot, synthesized result with 95% CI for each subgroup has been visualized on the right. MACE, major adverse cardiovascular events; EPA, eicosapentaenoic acids; DHA, docosahexaenoic acids; HR, hazard ratio; 95% CI, 95% confidence interval.

Thirteen studies [7, 9, 12, 13, 14, 15, 16, 17, 18, 21, 22, 23, 24] with 75,611 participants (37,804 in the n-3 PUFA group and 37,807 in the control group) on MACE outcome showed risks for MACE could not be significantly reduced by n-3 PUFA (HR: 0.98, 95% CI: 0.91–1.06; *p* = 0.592) with significant heterogeneity (*$I^{2}$* = 62.7%; *p* = 0.001) (Fig. 2A). After removing one heterogeneous study [22] (8179 participants), the HR turned to 1.00 (95% CI: 0.96–1.05; *p* = 0.872) with little heterogeneity (*$I^{2}$* = 0.0%; *p* = 0.955). In subgroup analysis, n-3 PUFA could not significantly reduce MACE on the prevention type (secondary prevention (6 [6, 7, 13, 14, 15, 24]): 1.07, 95% CI (0.97–1.18); mixed prevention (n = 6 [12, 16, 17, 18, 22, 23]): 0.92, 95% CI (0.82–1.04); only one study [21] for primary prevention: (0.97, 95% CI: 0.87–1.08), statin use proportion in trial (<50% (n = 4 [7, 9, 12, 18]): 0.99, 95% CI: 0.90–1.09; ≥50% (n = 8 [13, 14, 15, 16, 21, 22, 23, 24]): 0.98, 95% CI: 0.89–1.09), antiplatelet drug use proportion in trial (<50% (n = 3 [7, 18, 21]): 0.98, 95% CI: 0.91–1.05; ≥50% (n = 8 [9, 13, 14, 15, 16, 17, 23, 24]): 1.02, 95% CI: 0.96–1.07) or actual n-3 PUFA intake amount (<2 g/d (n = 6 [9, 15, 16, 17, 18, 21]): 1.00, 95% CI: 0.96–1.05; ≥2 g/d (n = 5 [7, 12, 22, 23, 24]): 0.93, 95% CI: 0.78–1.12). Only one study [22] was included for EPA use analysis (HR: 0.75, 95% CI: 0.68–0.83). Therefore, more evidence was still required for this result (Fig. 2B). Thank you.

### 3.3 MI

**Fig. 3.**
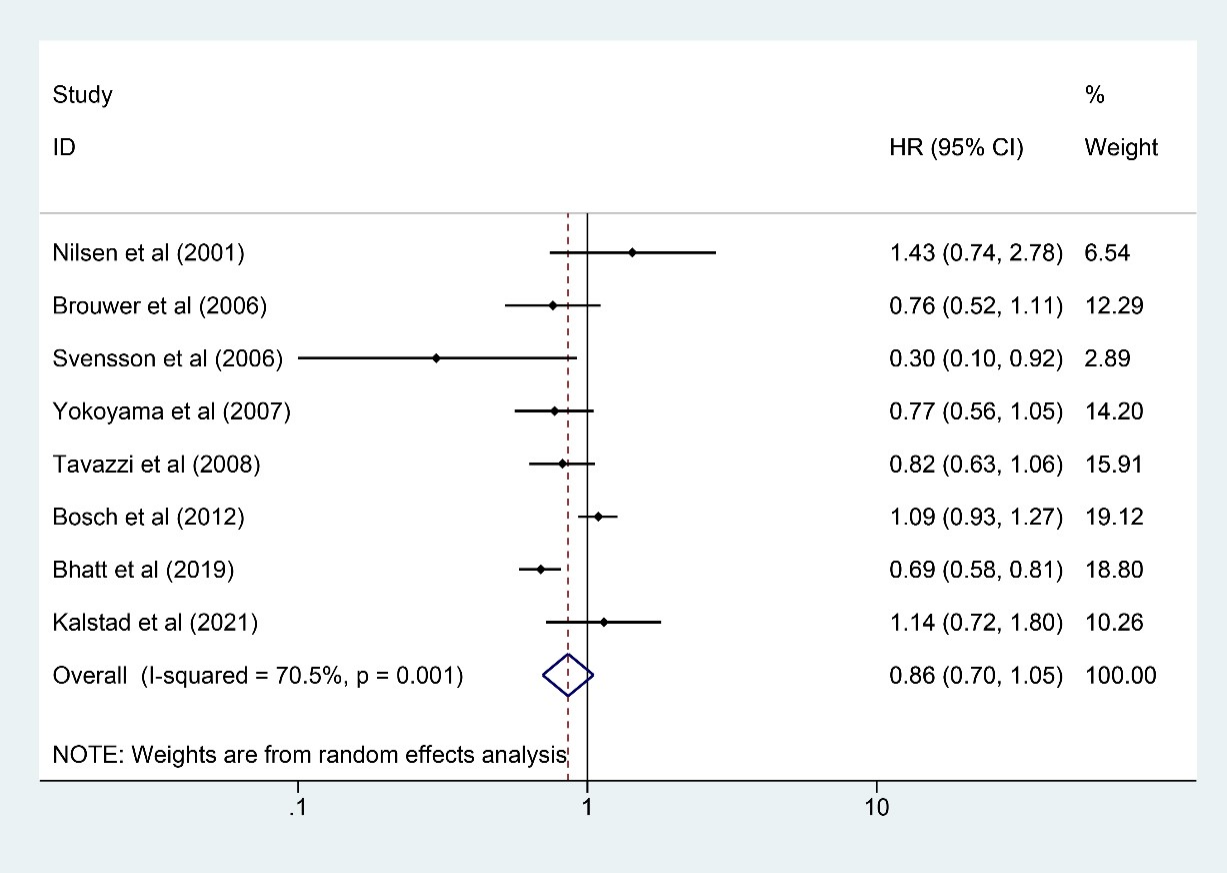
**Forest plot for MI**. Forest plot of main result on MI. Each bar and the middle diamond represented HR and 95% CI for each included study with the detailed value marked on right. The bottom diamond represented the synthesized result, if the whole diamond located on the left of vertical full line (on 1.00), the risk of MI was significantly reduced, if the whole diamond located on the right of vertical full line, the risk of MI was significantly improved, otherwise there was no statistically significant association. Vertical dashed line in red represented location of synthesized HR from which we could presume the trend of the association. MI, myocardial infarction; HR, hazard ratio; 95% CI, 95% confidence interval.

Eight studies [7, 8, 9, 10, 11, 16, 22, 24] involving 48,401 participants (24,221 in the n-3 PUFA group and 24,180 in the control group) reported MI outcome. The results indicated that the existence of risks for MI could not be significantly reduced by n-3 PUFA (HR: 0.86, 95% CI: 0.70–1.05; *p* = 0.137) with significant heterogeneity (*$I^{2}$* = 70.5%; *p* = 0.001) (Fig. 3). After removing 3 heterogeneous studies [9, 16, 22] (20,921 participants), the HR kept stable from 0.86 (95% CI: 0.70–1.05; *p* = 0.137) to 0.86 (95% CI: 0.72–1.03; *p* = 0.112) with a shortened confidence interval and little heterogeneity (*$I^{2}$* = 15.5%; *p* = 0.316). Main heterogeneity across analysis on MI was found among the three studies [9, 16, 22].

### 3.4 CHD

**Fig. 4.**
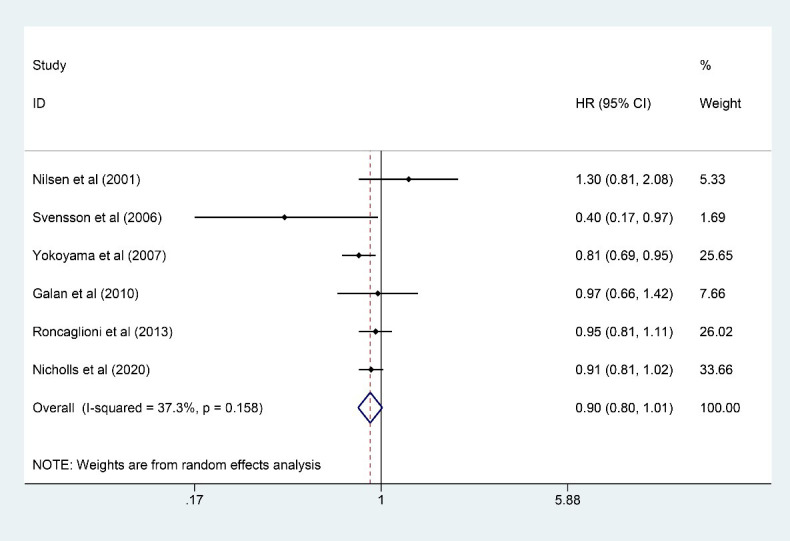
**Forest plot for CHD**. Forest plot of main result on CHD. Each bar and the middle diamond represented HR and 95% CI for each included study with the detailed value marked on right. The bottom diamond represented the synthesized result, if the whole diamond located on the left of vertical full line (on 1.00), the risk of CHD was significantly reduced, if the whole diamond located on the right of vertical full line, the risk of CHD was significantly improved, otherwise there was no statistically significant association. Vertical dashed line in red represented location of synthesized HR from which we could presume the trend of the association. CHD, coronary heart disease; HR, hazard ratio; 95% CI, 95% confidence interval.

Six studies [7, 9, 10, 13, 18, 23] involving 47,243 participants (23,615 in the n-3 PUFA group and 23,628 in the control group) reported results on CHD, and n-3 PUFA had the trend to reduce the incidence of CHD, but the statistic was not significant (HR: 0.90, 95% CI: 0.80–1.01; *p* = 0.079) with little heterogeneity (*$I^{2}$* = 37.3%; *p* = 0.158) (Fig. 4). Little intra-study heterogeneity restricted the implementation of sensitivity analysis.

### 3.5 Revascularization

**Fig. 5.**
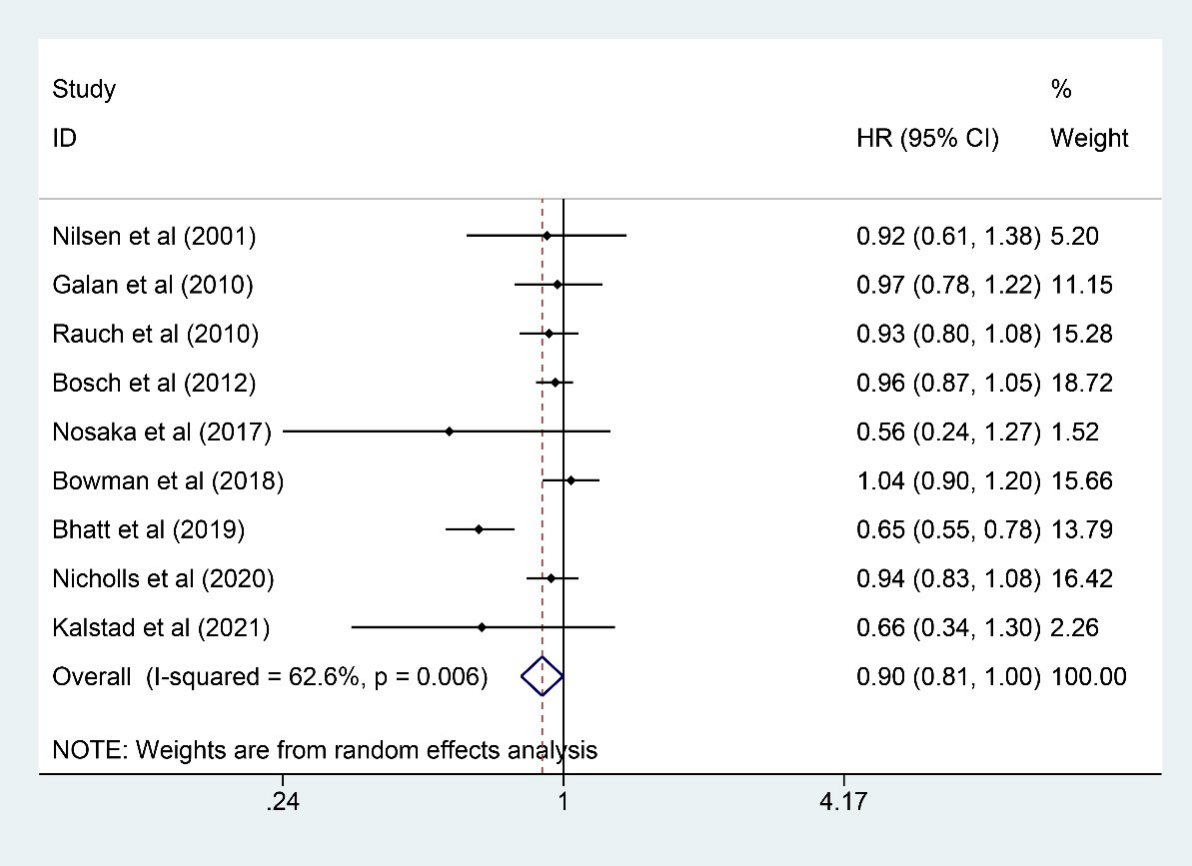
**Forest plot for revascularization**. Forest plot of main result on revascularization. Each bar and the middle diamond represented HR and 95% CI for each included study with the detailed value marked on right. The bottom diamond represented the synthesized result, if the whole diamond located on the left of vertical full line (on 1.00), the risk of revascularization was significantly reduced, if the whole diamond located on the right of vertical full line, the risk of revascularization was significantly improved, otherwise there was no statistically significant association. Vertical dashed line in red represented location of synthesized HR from which we could presume the trend of the association. HR, hazard ratio; 95% CI, 95% confidence interval.

Nine studies [7, 13, 15, 16, 20, 21, 22, 23, 24] were included for analysis on revascularization with a total of 57,144 participants analyzed (28,601 in the n-3 PUFA group and 28,543 in the control group). N-3 PUFA could significantly reduce the incidence of revascularization (HR: 0.90, 95% CI: 0.81–1.00; *p* = 0.006), although the upper 95% CI was on 1.00, and the *p* value for HR was 0.006 (<0.05). Significant heterogeneity was observed (*$I^{2}$* = 62.6%; *p* = 0.006) (Fig. 5). After removing three heterogeneous studies [20, 22, 24] (3431 participants), the HR changed from 0.90 (95% CI: 0.81–1.00; *p* = 0.006) to 0.96 (95% CI: 0.91–1.02; *p* = 0.221) with little heterogeneity observed (*$I^{2}$* = 0.0%; *p* = 0.914). Synthesized results on revascularization were not robust since the results changed after heterogeneous studies were removed. In this case, more relevant studies are still needed to confirm the controversial results.

### 3.6 Stroke

**Fig. 6.**
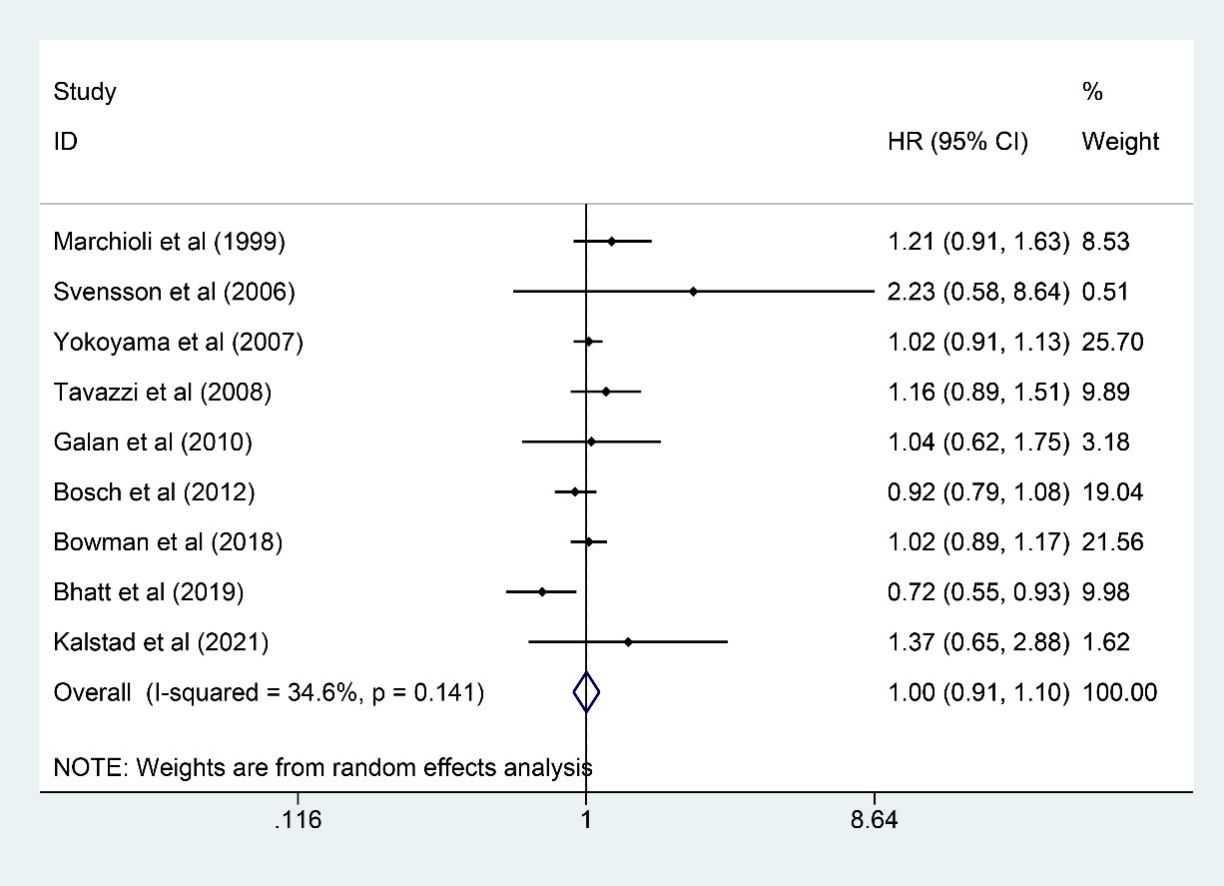
**Forest plot for stroke**. Forest plot of main result on stroke. Each bar and the middle diamond represented HR and 95% CI for each included study with the detailed value marked on right. The bottom diamond represented the synthesized result, if the whole diamond located on the left of vertical full line (on 1.00), the risk of stroke was significantly reduced, if the whole diamond located on the right of vertical full line, the risk of stroke was significantly improved, otherwise there was no statistically significant association. Vertical dashed line in red represented location of synthesized HR from which we could presume the trend of the association. HR, hazard ratio; 95% CI, 95% confidence interval.

Nine trials [6, 9, 10, 11, 13, 16, 21, 22, 24] involving 76,860 participants (38,457 and 38,403 in n-3 PUFA and control groups, respectively) were eligible for stroke outcome analysis, and n-3 PUFA exerted little effect on reducing stroke incidence (HR: 1.00, 95% CI: 0.91–1.10; *p* = 0.967) with little heterogeneity (*$I^{2}$* = 34.6%; *p* = 0.141) (Fig. 6).

### 3.7 SCD

**Fig. 7.**
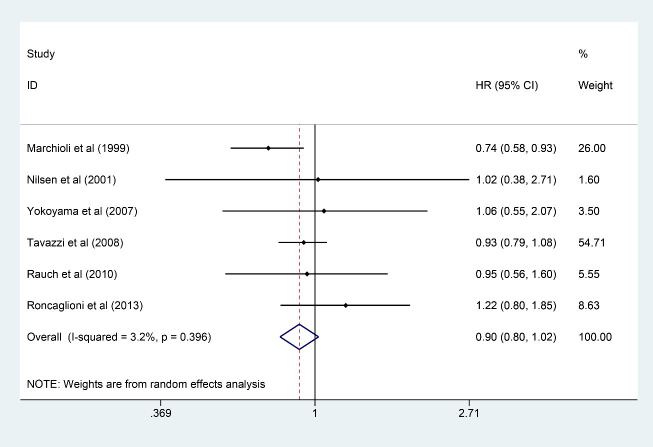
**Forest plot for SCD**. Forest plot of main result on SCD. Each bar and the middle diamond represented HR and 95% CI for each included study with the detailed value marked on right. The bottom diamond represented the synthesized result, if the whole diamond located on the left of vertical full line (on 1.00), the risk of SCD was significantly reduced, if the whole diamond located on the right of vertical full line, the risk of SCD was significantly improved, otherwise there was no statistically significant association. Vertical dashed line in red represented location of synthesized HR from which we could presume the trend of the association. SCD, sudden cardiac death; HR, hazard ratio; 95% CI, 95% confidence interval.

Six studies [6, 7, 10, 11, 15, 18] involving 53,575 participants were analyzed for SCD (26,805 and 26,770 in the n-3 PUFA group and the control group, respectively). It was found that n-3 PUFA could not improve the outcome of SCD (HR: 0.90, 95% CI: 0.80–1.02; *p* = 0.111) with little heterogeneity (*$I^{2}$* = 3.2%; *p* = 0.396) (Fig. 7).

### 3.8 CV Mortality

**Fig. 8.**
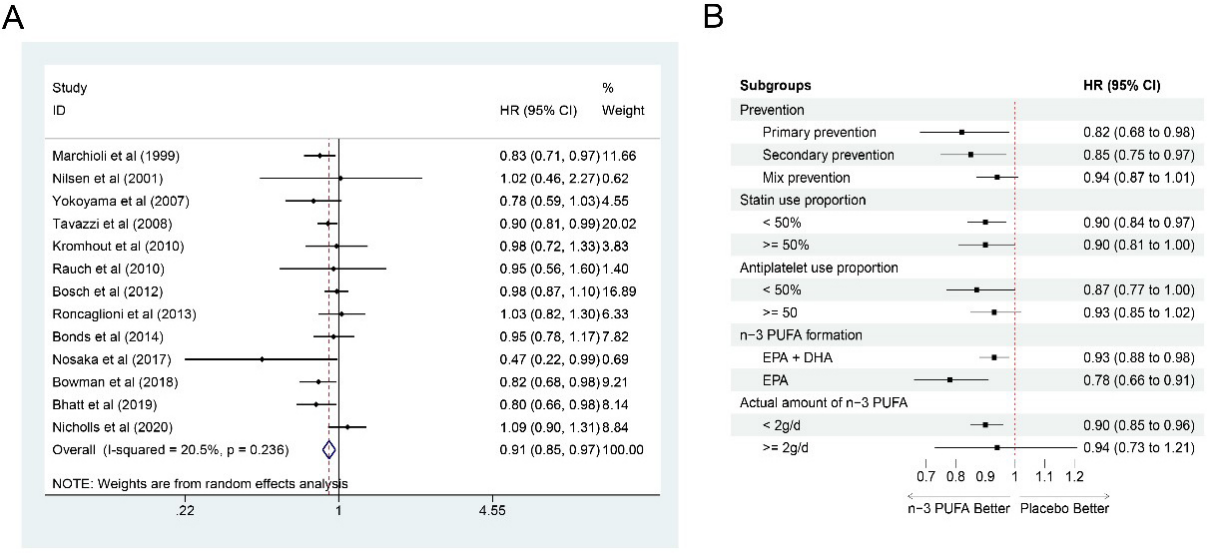
**Forest plot for CV mortality**. (A) Forest plot of main result on CV mortality. Each bar and the middle diamond represented HR and 95% CI for each included study with the detailed value marked on right. The bottom diamond represented the synthesized result, if the whole diamond located on the left of vertical full line (on 1.00), the risk of CV mortality was significantly reduced, if the whole diamond located on the right of vertical full line, the risk of CV mortality was significantly improved, otherwise there was no statistically significant association. Vertical dashed line in red represented location of synthesized HR from which we could presume the trend of the association. (B) Forest plot of subgroup analysis. Each outcome included a couple of subgroups, the reference list of studies in each subgroup has been marked in the forest plot, synthesized result with 95% CI for each subgroup has been visualized on the right. EPA, eicosapentaenoic acids; DHA, docosahexaenoic acids; HR, hazard ratio; 95% CI, 95% confidence interval.

Thirteen studies [6, 7, 10, 11, 14, 15, 16, 18, 19, 20, 21, 22, 23] were selected to calculate CV mortality. Totally, 111,082 participants were included, among which, 56,051 were in the n-3 PUFA group and 55,031 in the control group. The synthesized results showed that n-3 PUFA intake could significantly reduce CV mortality (HR: 0.91, 95% CI: 0.85–0.97; *p* = 0.003) with little heterogeneity (*$I^{2}$* = 20.5%; *p* = 0.236) (Fig. 8A). In subgroups in terms of secondary prevention (HR: 0.85, 95% CI: 0.75–0.97; n = 5 [6, 7, 14, 15, 20]), <50% (HR: 0.90, 95% CI: 0.84–0.97; n = 5 [6, 7, 11, 18, 19]) and ≥50% (HR: 0.90, 95% CI: 0.81–1.00; n = 8 [10, 14, 15, 16, 20, 21, 23]) population with statin use, <50% population with antiplatelet use (HR: 0.87, 95% CI: 0.77–1.00; n = 4 [7, 10, 18, 21]), EPA + DHA intake (HR: 0.93, 95% CI: 0.88–0.98; n = 10 [6, 7, 11, 14, 15, 16, 18, 19, 21, 23]), only EPA intake (HR: 0.78, 95% CI: 0.66–0.91; n = 3 [10, 20, 22]), and <2 g/d n-3 PUFA intake (HR: 0.90, 95% CI: 0.85–0.96; n = 7 [6, 10, 11, 15, 16, 18, 21]), n-3 PUFA could significantly reduce CV mortality. No statistical significance was observed in other subgroups (Fig. 8B), and most results of subgroup analyses were consistent with the total result on CV mortality.

### 3.9 All-Cause Mortality

**Fig. 9.**
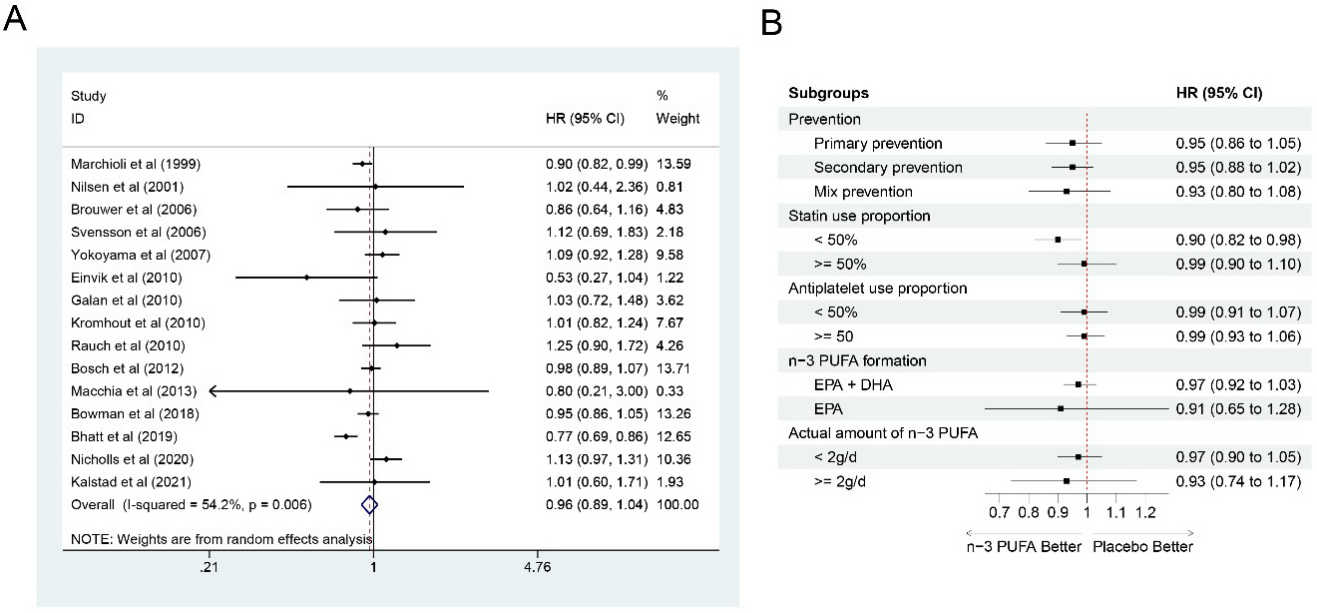
**Forest plot for all-cause mortality**. (A) Forest plot of main result on all-cause mortality. Each bar and the middle diamond represented HR and 95% CI for each included study with the detailed value marked on right. The bottom diamond represented the synthesized result, if the whole diamond located on the left of vertical full line (on 1.00), the risk of all-cause mortality was significantly reduced, if the whole diamond located on the right of vertical full line, the risk of all-cause mortality was significantly improved, otherwise there was no statistically significant association. Vertical dashed line in red represented location of synthesized HR from which we could presume the trend of the association. (B) Forest plot of subgroup analysis. Each outcome included a couple of subgroups, the reference list of studies in each subgroup has been marked in the forest plot, synthesized result with 95% CI for each subgroup has been visualized on the right. EPA, eicosapentaenoic acids; DHA, docosahexaenoic acids; HR, hazard ratio; 95% CI, 95% confidence interval.

There were 15 studies [6, 7, 8, 9, 10, 12, 13, 14, 15, 16, 17, 21, 22, 23, 24] involving 93,613 participants (46,825 in the n-3 PUFA group and 46,788 in the control group) included for the meta-analysis for all-cause mortality, and n-3 PUFA could not significantly reduce the risk for all-cause mortality (HR: 0.96, 95% CI: 0.89–1.04; *p* = 0.339). Significant heterogeneity was found (*$I^{2}$* = 54.2%; *p* = 0.006) (Fig. 9A). After removing three heterogeneous studies [12, 22, 24] (9756 participants), the HR turned to 0.98 (95% CI: 0.94–1.04; *p* = 0.544) with little heterogeneity (*$I^{2}$* = 8.4%; *p* = 0.363) and a shortened confidence interval. Results on all-cause mortality kept robust through sensitivity analysis. As for subgroup analysis, on trials of <50% population with statin use (HR: 0.78, 95% CI: 0.66–0.91; n = 5 [6, 7, 8, 9, 12]), n-3 PUFA could reduce the risk for all-cause mortality. However, statistics was not significant in other groups (Fig. 9B).

### 3.10 Hospitalization

Two studies [11, 17] involving a total of 7571 participants were included in analysis on hospitalization (3783 in the n-3 PUFA group and 3788 in the control group), and n-3 PUFA could not significantly reduce the hospitalization incidence (HR: 0.99, 95% CI: 0.81–1.20; *p* = 0.884) with little heterogeneity found (*$I^{2}$* = 33.1%; *p* = 0.221) (**Supplementary Fig. 1**).

### 3.11 Hospitalization for All Heart Disease

Five studies [11, 16, 18, 20, 22] were obtained to synthesize the results on hospitalization for all heart diseases. In detail, 40,441 participants were totally included, with 20,227 in the n-3 PUFA group and 20,214 in the control group. N-3 PUFA presented the signal to reduce the risk for hospitalization for all heart diseases (HR: 0.91, 95% CI: 0.83–1.00; *p* = 0.059) with significant heterogeneity (*$I^{2}$* = 70.9%; *p* = 0.008), but the statistic was not significant (**Supplementary Fig. 2**). After removing two heterogeneous studies [20, 22] (8417 participants), the HR turned to 0.96 (95% CI: 0.92–1.00; *p* = 0.048) with little heterogeneity (*$I^{2}$* = 0.0%; *p* = 0.475). More evidence on the outcome of hospitalization for all heart diseases is still needed, because the upper 95% CIs are on 1.00 and the *p* values for HR are close to 0.05.

### 3.12 Hospitalization for Heart Failure

Six studies [11, 16, 17, 20, 23, 24] reported outcomes on hospitalization for heart failure. A total of 34,427 participants, with 17,227 in the n-3 PUFA group and 17,200 in the control group, were analyzed, and the integrated results showed that n-3 PUFA could not decrease the incidence for hospitalization for heart failure (HR: 0.97, 95% CI: 0.91–1.04; *p* = 0.450) with little heterogeneity (*$I^{2}$* = 0.0%; *p* = 0.715) (**Supplementary Fig. 3**).

### 3.13 AF

Three studies [6, 23, 24] with 14,678 participants (7333 in the n-3 PUFA group and 7345 in the control group) reported the AF results, and the incidence of AF was significantly increased with n-3 PUFA intake (HR: 1.56, 95% CI: 1.27–1.91; *p *< 0.001) with little heterogeneity (*$I^{2}$* = 0.0%; *p* = 0.407) (**Supplementary Fig. 4**).

### 3.14 Publication Bias

No publication biases were found across analyses on MACE (Begg’s test *p* = 0.428, Egger’s test *p* = 0.427), MI (Begg’s test *p* = 1.000, Egger’s test *p* = 0.776), CHD (Begg’s test *p* = 1.000, Egger’s test *p* = 0.858), revascularization (Begg’s test *p* = 0.048, Egger’s test *p* = 0.282), stroke (Begg’s test *p* = 0.348, Egger’s test *p* = 0.451), SCD (Begg’s test *p* = 1.000, Egger’s test *p* = 0.510), CV mortality (Begg’s test *p* = 0.760, Egger’s test *p* = 0.532), and all-cause mortality (Begg’s test *p* = 0.428, Egger’s test *p* = 0.598).

## 4. Discussion

Considerable interest has been focused on potential protection from n-3 PUFA on CVD. Omega-3 PUFA supplements confer favorable effects on lipoprotein metabolism and inflammatory, oxidative, thrombotic, vascular, and arrhythmogenic factors existing in CVD [37, 38]. Marine-derived n-3 PUFA has been investigated for decades in patients with CVD or in patients with high-risk factors for CVD, yielding conflicting results on its effects on CV events. In current systematic review and meta-analysis, we included 19 RCTs with 116,498 populations taking n-3 PUFA or placebo. We found n-3 PUFA intake could significantly reduce the risk for revascularization and CV mortality, however, increased the risk for AF. No significant effects were observed with respect to MACE, MI, CHD, SCD, all-cause mortality, hospitalization, hospitalization for all heart disease and hospitalization for heart failure. Before clinical practice, medical caregivers should balance the benefits and harm of n-3 PUFA for CVD prevention and treatment.

In 2017, a scientific statement was designed by the AHA to assess the impact of supplements with n-3 PUFA on CVD based on several RCTs and meta-analyses, which confirmed that no consensus was achieved on n-3 PUFA intake for the prevention of CVD in populations with high-risk factors for CVD (class III, B), and that taking n-3 PUFA for the secondary prevention of CHD death was reasonable in people with diagnosed CHD (class IIa, A) [39]. No clear and beneficial effects of n-3 PUFA on MACE and CHD were revealed in the current meta-analysis, and no analysis about CHD death was conducted because existing data and evidence were limited on that outcome. In subgroup analysis, intake of EPA only had a strong effect on reducing MACE and CV mortality, and the finding was consistent with previous studies. Two previous clinical trials have revealed the potential benefit of purified formulations of EPA alone [22, 40]. The open-label JELIS study prescribed 1.8 g/d EPA in combination with a statin for a median of 4.6 yr follow-up in 18,645 Japanese patients with hypercholesterolemia, which resulted in fewer CHD events compared with statin therapy alone (2.8% vs. 3.5%; HR: 0.81; 95% CI: 0.69–0.95) [40]. The JELIS trial incorporated patients with a mean low density lipoprotein cholesterol (LDL-C) level of 180 mg/dL, but these patients were treated with a rather low level of statins (pravastatin 10 mg or simvastatin 5 mg), and revascularization was included in a broad composite clinical endpoint [13]. In another trial of REDUCE-IT, the primary authors reported an administration of 4 g/d EPA compared with mineral oil for a median duration of 4.9 yr follow-up in 8179 statin-treatment patients with a high triglyceride level between 135–499 mg/dL. The CV events were significantly reduced in the EPA group (17.2% vs. 22.0%; HR: 0.75, 95% CI: 0.68–0.83) [22]. Additional analyses of both EPA intake studies suggested an inverse association between plasma EPA concentration during treatment and the rate of CV events [41].

Early RCTs in the 1990s have suggested cardiovascular benefits of n-3 PUFA after an acute myocardial infarction (AMI). The DART randomized trial demonstrated a 29% reduction in 2-yr mortality in patients randomized to eat fatty fish twice per week [42]. The GISSI trial indicated a 21% reduction in all-cause mortality and a 45% reduction in SCD in patients administrated with 850 mg EPA/DHA compared to placebo for 3.5 yr follow-up period [6]. Another three clinical trials administrated with EPA + DHA from 400–840 mg/d presented insignificant results [13, 14, 15]. Similarly, no risk reduction was observed by 1 g/d EPA + DHA intake in diabetic patients free of CVD in the ASCEND trial [21]. Given that the results on this topic are controversial in RCTs and meta-analysis, a new meta-analysis was conducted with all eligible RCTs included, and comprehensive cardiovascular outcomes were provided. After comparing the included studies, the inconsistent results had some possible sources: n-3 PUFA was used for secondary prevention on CVD in some studies, and considerable statins and antiplatelet drugs capable of influencing the efficacy of n-3 PUFA were used in the treatment process. Additionally, different amounts of n-3 PUFA administrated and different demographic baseline characteristics, such as populations with different risk factors, would also have interactions with n-3 PUFA intake.

The use of n-3 PUFA was observed to potentially prevent the risk of CV mortality. However, little impact was noted on all-cause mortality. The protection derived from n-3 PUFA on CV mortality could be explained by the low dose of n-3 PUFA intake controlling SCD through an antiarrhythmic effect [43]. Sensitivity analysis on CV mortality showed consistent negative results, which could be explained by the high proportion of death caused by cardiac reasons. Besides, supplements with n-3 PUFA failed to reduce the risk of MI and stroke, which could be influenced by the dose and duration of n-3 PUFA intake. Results from subgroup analysis showed a significantly reduced risk for CV mortality in n-3 PUFA use for secondary prevention, suggesting that populations exposed to higher CV risks seemed to benefit most from n-3 PUFA. The above hypothesis could also be supported by a previous meta-analysis claiming that benefits from n-3 PUFA to reduce CHD were more evident in participants with elevated triglyceride or elevated LDL-C levels [44]. In the FOURIER trial, MACE was observed in 14.4% of diabetic patients after 36 months of statin-PCSK9 inhibitors therapy [45]. Besides, the possible beneficial effects of n-3 PUFA were merely more likely to be detectable because of a greater number of CV events. Similar results were also detected in the JELIS trial [10].

Additionally, there are also previous meta-analyses on this controversial topic (Table 3, Ref. [6, 7, 8, 9, 12, 14, 17, 19, 20, 21, 32, 46]). Casula *et al*. [32] included 16 RCTs involving 81,073 participants and revealed risk of CV mortality, MACE and MI could be significantly reduced by n-3 PUFA intake. To our best knowledge, results on CV mortality, MACE and MI were likely to be influenced by the dosage and duration of n-3 PUFA intake, and results on MACE were not stable in the study of Casula *et al*. [32] because the 95% CI was close 1. With more updated results included, the synthesized results might change. Additionally, sensitivity analysis, publication bias, source of heterogeneity and study limitation were not well presented or discussed, which was thought important to provide informative findings. This study included 19 RCTs involving 116,498 participants, and findings from the current study were stronger and more powerful. Yan *et al*. [46] included 15 RCTs with 141,164 participants, and 10 CVD related outcomes were mainly reported. However, hospitalization for all heart diseases and hospitalization for heart failure were absent. They revealed that n-3 PUFA could reduce MACE, MI and CV mortality, while publication bias was not reported, and the synthesized results might not be as stable as expected. Yan *et al*. [46] also performed meta-analysis on bleeding events and cancer. However, the fact was that only few included studies provided outcomes on bleeding events or disorders, and that significant heterogeneity was also observed across studies reporting bleeding events. Incidence of cancer should not be listed as a main aim in a meta-analysis focusing on CVD outcomes, because the authors of cardiologists might not be so familiar with cancer outcomes as CVD outcomes. Thus, evaluation on cancer incidence was questionable, and all these concerns from Yan *et al*. [46] needed to be addressed with more high-quality evidence.

**Table 3.** **The comparison to previous meta-analyses**.

	Casula *et al*. [32] (PMID: 32634581)	Yan *et al*. [46] (PMID: 36103100)	Current study
Year	2020	2022	2022
Published journal	Pharmacological Research (ISSN: 1043-6618)	Cardiovascular Drugs and Therapy (ISSN: 0920-3206)	Reviews in Cardiovascular Medicine (ISSN: 1530-6550)
Populations	81,073 participants, mean age from 49-74 year	141,164 participants	116,498 participants
N-3 PUFA intake	EPA + DHA or EPA	EPA + DHA or EPA	EPA + DHA or EPA
Prevention type	secondary or mixed	secondary or mixed	secondary or mixed
Included study type	RCTs	RCTs	RCTs
Included study number	16	15	19
Included study quality	all trials are of high quality (assessed by Jadad score)	all trials are of high quality	Nilsen *et al*. [7] (2001) and Einvik *et al*. [12] (2010) were lack of detailed descriptions on blinding methods. Nilsen *et al*. [7] (2001) failed to provide evidence to support the process of randomization and allocation concealment; allocation concealment was also absent in Einvik *et al*. [12] (2010). Bowman *et al*. [21] (2018) was an open-label study that did not take blinding for participants and outcome assessments. Incomplete outcome data existed in Brouwer *et al*. [8] (2006), Kromhout *et al*. [14] (2010) and Bonds *et al*. [19] (2014). There was unclear for other source of bias in Marchioli *et al*. [6] (1999), Svensson *et al*. [9] (2006), Einvik *et al*. [12] (2010), Macchia *et al*. [17] (2013), Bonds *et al*. [19] (2014) and Nosaka *et al*. [20] (2017)
Analyzed outcomes	6 outcomes (all-cause mortality, CV mortality, no CV mortality, MACE, MI and stroke)	10 outcomes (MACE, MI, HF, stroke, AF, CV mortality, all-cause mortality, gastrointestinal problems, bleeding-related disorders and cancer)	12 outcomes (MACE, MI, CHD, revascularization, stroke, SCD, CV mortality, all-cause mortality, hospitalization, hospitalization for all heart disease, hospitalization for heart failure and AF)
Total findings	risk of CV mortality, MACE and MI was reduced	risk of MACE, MI and CV mortality was reduced; risk of AF was increased	risk of revascularization and CV mortality was reduced; risk of AF was increased
Sensitivity analysis	not reported	performed on MACE, MI, HF, AF, stroke, all-cause mortality and cancer. Results on MI changed	performed on MACE, MI, revascularization, all-cause mortality, hospitalization for all heart disease. Results on revascularization changed
Findings from subgroup analysis	(1) risk of CV mortality and MI was reduced in secondary prevention trials; (2) risk reduction on CV mortality, MACE and MI was effective for more than 1 g/d n-3 PUFA intake; (3) EPA + DHA was only effective on CV mortality over EPA	(1) only EPA seemed to be more effective than EPA + DHA; (2) risk of MACE was reduced in secondary prevention trials; (3) risk of MI was reduced in primary prevention trials; (4) risk of stroke patients with MI was increased; (5) EPA was associated with the risk of bleeding	(1) n-3 PUFA was not associated with MACE outcomes across subgroup analyses; (2) risk of CV mortality was reduced across subgroup analyses except mixed prevention trials, and ≥2 g/d n-3 PUFA intake trials; (3) risk of all-cause mortality was reduced in trials with statin use in <50% populations
Heterogeneity	not reported	(1) no significant heterogeneity: stroke, CV mortality, cancer; (2) mild heterogeneity on MI, HF; (3) slight heterogeneity on all-cause mortality; (4) moderate heterogeneity on MACE, AF, bleeding-related disorders; (5) significant heterogeneity on gastrointestinal problems	(1) little heterogeneity: CHD, stroke, SCD, CV mortality, hospitalization, hospitalization for all heart disease, AF; (2) significant heterogeneity: MACE, MI, revascularization; all-cause mortality
Publication bias	not reported	not reported	no publication bias on MACE, MI, CHD, revascularization, stroke, SCD, CV mortality and all-cause mortality
Conclusion	n-3 PUFA significantly improves cardiovascular outcomes, with higher benefit in secondary CV prevention, using more than 1 g/d and taking EPA alone	n-3 PUFA may reduce risk of MACE, MI, CV mortality. EPA alone seems to be effective. N-3 PUFA dose not increase gastrointestinal problems, bleeding-related disorders, or cancer	n-3 PUFA could reduce the risk of CV mortality and revascularization, it also increases the AF incidence; the benefits and harm of n-3 PUFA should be balanced when using for patients or high risk populations
Study limitation	not reported	discussion on dietary supplements type was lack; heterogeneity among included studies; limitations from JELIS and REDUCE-IT study; small number of studies on AF outcome	inconsistent outcome definitions; high heterogeneity across some analyses; inconsistent n-3 PUFA formation; small number of studies on AF and hospitalization

Abbreviations: MACE, major adverse cardiovascular events; MI, myocardial infarction; HF, heart failure; CHD, coronary heart disease; SCD, sudden cardiac death; AF, atrial fibrillation; CVD, cardiovascular disease; n-3 PUFA, Omega-3 polyunsaturated fatty acids; EPA, eicosapentaenoic acids; DHA, docosahexaenoic acids; RCT, randomized controlled trial.

Current meta-analysis is endowed with some merits compared to previous meta-analysis. First of all, this meta-analysis is the most comprehensive and timely-updated study up till now. Totally, 19 related RCTs with 116,498 populations were included, and 12 outcomes of interest on CVD were also specially analyzed. Generally speaking, abundant evidence will always bring robust and reliable conclusions, and the pooled CVD outcomes will take more insights for clinical practice. Then, detailed subgroup analyses were performed on variables about statin use and antiplatelet drug use, and the results of the subgroup analysis also supported that n-3 PUFA seemed to be more effective and might bring more benefits to populations at a high risk for potential CV events. There were also several limitations on current meta-analysis. Firstly, definitions of the outcome of interest in included studies were not consistent, which might lead to some biases in the pooled results and potential heterogeneity among the included studies for analysis. Second, heterogeneity seemed to be high in some analyses. In this case, all analyses were performed using random-effects models, and sensitivity analyses were performed on the outcome of interest with significant heterogeneity. Third, n-3 PUFA formation was not consistent in the included studies, which potentially contributed to some biased stuff in the analysis. Given that, additional subgroup analysis was performed based on different n-3 PUFA intake formations. Finally, although 12 outcomes of interest were pre-set for clinical reference, the study number for AF and hospitalization was relatively limited. Thus, more evidence on the outcomes of AF and hospitalization with n-3 PUFA intake was still required to confer robust and conclusive results in the future.

## 5. Conclusions

In this meta-analysis, n-3 PUFA intake is found to significantly reduce the risk for revascularization and CV mortality, however, it also increases the risk for AF. Besides, n-3 PUFA seems to be more effective on populations with CV events for secondary prevention, especially with EPA intake only. Neutral results are observed with respect to MACE, MI, CHD, SCD, all-cause mortality, hospitalization, hospitalization for all heart disease and hospitalization for heart failure. Before clinical practice, medical caregivers should balance the benefits and harm of n-3 PUFA for CVD prevention and treatment.

## Supplementary Material

Supplementary material associated with this article can be found, in the online version, at https://doi.org/10.31083/j.rcm2401024.
