# Supplementary material for: Omega-3 Polyunsaturated Fatty Acids Supplements and Cardiovascular Disease Outcome: A Systematic Review and Meta-Analysis on Randomized Controlled Trials
Source: Rev Cardiovasc Med. 2023 Jan 12;24(1):24. doi: 10.31083/j.rcm2401024 (PMC11270471; doi:10.31083/j.rcm2401024)
Supplement: Supplementary file 1 [file 2153-8174-24-1-024-s1.zip › Supplementary Tables/Table S2. Study Protocol.pdf]

INPLASY PROTOCOL

To cite: Qi et al. Omega-3 polyunsaturated fatty acids supplements and cardiovascular disease outcome: A systematic review and meta-analysis on randomized controlled trials. Inplasy protocol 2022110027. doi: 10.37766/inplasy2022.11.0027

Received: 06 November 2022

Published: 06 November 2022

Corresponding author:
Hao Huang

huanghaosankang@126.com

Author Affiliation:
Department of Critical Rehabilitation, Shanghai Third Rehabilitation Hospital.

Support: **There is no financial support.**

Review Stage at time of this submission: **Formal screening of search results.**

Conflicts of interest:
None declared.

Omega-3 polyunsaturated fatty acids supplements and cardiovascular disease outcome: A systematic review and meta-analysis on randomized controlled trials

Qi, X[1]; Zhu, H[2]; Ya, R[3]; Huang, H[4].

Review question / Objective: **In current study, we aimed to: (1) conduct a systematic review and meta-analysis by incorporating all eligible RCTs; (2) report results on CVD outcomes in a more comprehensive manner; (3) analyze the influence of statin and antiplatelet drug use on final results.**
Information sources: **We reviewed databases of Pubmed, EMBASE, Cochrane Library and Web of Science for eligible studies from the inception to Aug-15-2022. The combined search strategy of relevant keywords and Medical Subject Headings (MeSH) terms used in current study are: "Omega-3 fatty acids", "docosahexaenoic acid", "DHA", "Eicosapentaenoic acid", "EPA", "cardiovascular disease", "cardiovascular events", "coronary heart disease", "myocardial infarction", "stroke" and "randomized controlled trial". No special restrictions were applied to language. Reference lists of the retrieved literature were also searched manually. Primary authors could be contacted if there were lack of necessary data. Study characteristics including trial register have been summarized into a Table in main body of text.**

INPLASY registration number: This protocol was registered with the International Platform of Registered Systematic Review and Meta-Analysis Protocols (INPLASY) on 06 November 2022 and was last updated on 06 November 2022 (registration number INPLASY2022110027).

INTRODUCTION

Review question / Objective: **In current study, we aimed to: (1) conduct a systematic review and meta-analysis by incorporating all eligible RCTs; (2) report results on CVD outcomes in a more comprehensive manner; (3) analyze the influence of statin and antiplatelet drug use on final results.**

Qi et al. Inplasy protocol 2022110027. doi:10.37766/inplasy2022.11.0027 Downloaded from https://inplasy.com/inplasy-2022-11-0027/

Rationale: Many meta-analyses and randomized controlled trials (RCTs) on the use of Omega-3 supplements for cardiovascular disease (CVD) have come to different outcomes. Besides, previous meta-analyses have missed some key RCTs on this topic.

Condition being studied: Omega-3 polyunsaturated fatty acids (n-3 PUFA) include α-linolenic acid (ALA), eicosapentaenoic acids (EPA), and docosahexaenoic acids (DHA). ALA is abundant in plant, EPA and DHA are abundant in marine animals. Fish oil stemmed from marine animals is also rich in EPA and DHA. Over the past several decades, numerous population-based epidemiological studies have delineated that higher fish oil intake in the diet can reduce the incidence of cardiovascular events (CV events). The American Heart Association (AHA) also recommended patients with coronary heart disease (CHD) take 1g/d of EPA and DHA supplements as directed by their physician. Two to four g/d EPA and DHA capsules are recommended for patients with hypertriglyceridemia (HTG) under the guidance of their family doctors for treatment. Therefore, n-3 PUFA is desired by patients with cardiovascular disease (CVD) to treat their disease and populations with high-risk factors to prevent CVD. Although there is abundant evidence, nevertheless, outcomes derived from current evidence are inconsistent. From mechanistic aspects, n-3 PUFA confers protection against a wide range of CVD states including modulating cell membrane function, regulating cardiac rhythm, polishing endothelial function and inhibiting inflammatory, oxidative and thrombotic pathways implicated in atherosclerosis. N-3 PUFA also favors modulating triglyceride-rich lipoprotein metabolism. While in clinical aspects, there has been a great deal of controversy on the protective role of n-3 PUFA. Some clinical trials displayed a considerable beneficial profile of n-3 PUFA for reducing all-cause mortality, CV mortality, sudden cardiac death (SCD), CHD and stroke; while other clinical trials failed to confirm the protective effect. A recent meta-analysis on this similar topic included 16 randomized controlled trials (RCTs) and revealed n-3 PUFA could significantly improve CVD outcomes, especially for second prevention on 1g/d level with taking EPA only. To our great knowledge, that meta-analysis fails to report results on some other key CV outcomes for example hospitalization rate among participants, additionally, several important trials were not included. Importantly, no previous meta-analysis has ever analyzed the influence of statin and antiplatelet drug use on CVD outcomes with n-3 PUFA intake. Overall, these inconsistent results warrant a better understanding of the effects of n-3 PUFA on comprehensive subtypes of CVD states. Also, limitations of previous meta-analyses on the similar topic should be overcome and updated.

METHODS

Search strategy: We reviewed databases of Pubmed, EMBASE, Cochrane Library and Web of Science for eligible studies from the inception to Aug-15-2022. The combined search strategy of relevant keywords and Medical Subject Headings (MeSH) terms used in current study are: "Omega-3 fatty acids", "docosahexaenoic acid", "DHA", "Eicosapentaenoic acid", "EPA", "cardiovascular disease", "cardiovascular events", "coronary heart disease", "myocardial infarction", "stroke" and "randomized controlled trial". No special restrictions were applied to language. Reference lists of the retrieved literature were also searched manually.

Participant or population: Populations: Adult populations (≥18 yr) with CVD or with high-risk factors (e.g., smoking, obesity, lack of physical activity) for CVD. No restrictions on their gender, race, nationality and CV-related comorbidities (e.g., diabetes, hypertension, kidney circulation dysfunction).

Intervention: Intervention/comparison: Omega-3 PUFA from dietary supplements, capsules or drug prescriptions was used. Omega-3 PUFA directly achieved from

Qi et al. Inplasy protocol 2022110027. doi:10.37766/inplasy2022.11.0027 Downloaded from https://inplasy.com/inplasy-2022-11-0027/

marine fish food sources was not eligible because it was hard to quantify n-3 PUFA intake from this source.

Comparator: Intervention/comparison: Omega-3 PUFA from dietary supplements, capsules or drug prescriptions was used. Omega-3 PUFA directly achieved from marine fish food sources was not eligible because it was hard to quantify n-3 PUFA intake from this source.

Study designs to be included: Study design: Randomized controlled trial (RCT).

Eligibility criteria: All searched articles went into a two-step review process. They were initially screened for titles and abstracts, then the full texts of possibly eligible studies were reviewed by two independent authors. Any disagreements were resolved by a discussion in a group panel with another author who is of great knowledge of cardiology and evidence-based medicine.The eligible criteria following PICOS principles were listed:Populations: Adult populations (≥18 yr) with cardiovascular disease (CVD) or with high-risk factors (e.g., smoking, obesity, lack of physical activity) for CVD. No restrictions on their gender, race, nationality and CV-related comorbidities (e.g., diabetes, hypertension, kidney circulation dysfunction). Intervention/comparison: Omega-3 PUFA from dietary supplements, capsules or drug prescriptions was used. Omega-3 PUFA directly achieved from marine fish food sources was not eligible because it was hard to quantify n-3 PUFA intake from this source.Outcomes: At least one of the following outcomes reported with available data for calculating: major adverse cardiovascular events (MACE), CHD, revascularization, stroke, sudden cardiac death (SCD), CV mortality, all-cause mortality, hospitalization, hospitalization for all heart disease, hospitalization for heart failure and atrial fibrillation (AF). Study design: Randomized controlled trial (RCT) The same trial with longer follow-up period could be included to avoid duplication. Eligible RCTs should have registered protocol and provide ethics approval and consent of individuals. Observational studies, reviews, case reports, conference abstracts and experimental studies were excluded. Studies without essential data are also excluded.

Information sources: We reviewed databases of Pubmed, EMBASE, Cochrane Library and Web of Science for eligible studies from the inception to Aug-15-2022. The combined search strategy of relevant keywords and Medical Subject Headings (MeSH) terms used in current study are: "Omega-3 fatty acids", "docosahexaenoic acid", "DHA", "Eicosapentaenoic acid", "EPA", "cardiovascular disease", "cardiovascular events", "coronary heart disease", "myocardial infarction", "stroke" and "randomized controlled trial". No special restrictions were applied to language. Reference lists of the retrieved literature were also searched manually. Primary authors could be contacted if there were lack of necessary data. Study characteristics including trial register have been summarized into a Table in main body of text.

Main outcome(s): Major adverse cardiovascular events (MACE), CHD, revascularization, stroke, sudden cardiac death (SCD), CV mortality, all-cause mortality, hospitalization, hospitalization for all heart disease, hospitalization for heart failure and atrial fibrillation (AF).

Quality assessment / Risk of bias analysis: For evaluating the quality of included studies, we applied the Cochrane Risk of Bias Tool, which has been widely used for assessing the methodological quality of RCTs in meta-analyses. Seven specific bars in the Cochrane Risk of Bias Tool were objectively evaluated by two independent authors including the generation of randomized sequences, concealment of allocation protocols, blinding of study participants and related persons, blinding of outcome evaluators, incomplete data on study results, selective reporting of results and other sources of bias. If each bar from the Cochrane Risk of Bias Tool was not available or wrongly conducted, assessment on the bar would be high risk.

Qi et al. Inplasy protocol 2022110027. doi:10.37766/inplasy2022.11.0027 Downloaded from https://inplasy.com/inplasy-2022-11-0027/

Strategy of data synthesis: Fully adjusted HR and the corresponding 95% confidence intervals (95% CIs) for outcomes of interests obtained from Cox-Hazard regression analysis were mainly estimated with DerSimonian-Laird (D-L) random effects model because the assumptions could account for the presence of whining-study and between-study heterogeneity. The adjusted/unadjusted RR and OR in primarily included studies were approximately considered as HR. HRs and standard errors (SEs) originating from the correspondence 95% CIs were logarithmically transformed to stabilized variance, and then the distribution was normalized. Between-study heterogeneity was determined with the Cochran Q chi-square test and the I2. An I2 > 50% or a P value for the Q test < 0.1 was regarded as equal to significant heterogeneity. All analyses were performed using Stata software version 12.0 (https://www.stata.com/); two-sided P < 0.05 was statistically significant. When 95% CIs of HR were on 1.00, P value for HR would be checked of which P < 0.05 indicating statistical significance.

Subgroup analysis and Sensitivity analysis: Sensitivity analysis would be performed by removing one study each turn to reduce and elaborate the causes of the heterogeneity if significant heterogeneity was found. We would also conduct post-subgroup analyses to ascertain the influence of other risk factors on outcome results on MACE, CV mortality and all-cause mortality because there were abundant included studies on those outcomes. According to main characteristics of the populations and trial, the subgroups were identified as follows: the proportion of statin use populations (< 50% vs. ≥ 50%) in each trial, proportion of antiplatelet drug use populations (< 50% vs. ≥ 50%) in each trial, n-3 PUFA formulations (EPA + DHA vs. EPA) in each trial, actual amount of n-3 PUFA intake (< 2g/d vs. ≥ 2g/d) in each trial and prevention type (primary prevention vs. secondary prevention vs. mix prevention) in each trial. The subgroup analyses results were visualized by forest plots.

Language restriction: No restrictions.

Country(ies) involved: China.

Keywords: polyunsaturated fatty acids, cardiovascular disease, randomized controlled trial, meta-analysis.

Dissemination plans: Get the study published on high-impact journals, and update the systematic review and meta-analysis on time if available.

Contributions of each author:
Author 1 - Xue Qi - Xue Qi wrote the paper. Xue Qi, Hao Huang assume primary responsibility for the final content. All authors read and approved the final manuscript.
Email: qixuesankang@126.com
Author 2 - Hechen Zhu - Hao Huang, Hechen Zhu and Ru Ya helped design the study. Hechen Zhu and Ru Ya revised the statistical methodology. All authors read and approved the final manuscript.
Email: zhuhechenhuashan@126.com
Author 3 - Ru Ya - Hao Huang, Hechen Zhu and Ru Ya helped design the study. Hechen Zhu and Ru Ya revised the statistical methodology. All authors read and approved the final manuscript.
Author 4 - Hao Huang - Hao Huang, Hechen Zhu and Ru Ya helped design the study. Xue Qi, Hao Huang assume primary responsibility for the final content. All authors read and approved the final manuscript.
Email: huanghaosankang@126.com

Qi et al. Inplasy protocol 202211027. doi:10.37766/inplasy2022.11.0027 Downloaded from https://inplasy.com/inplasy-2022-11-0027/
