# Supplementary material for: Omega-3 Polyunsaturated Fatty Acids Supplements and Cardiovascular Disease Outcome: A Systematic Review and Meta-Analysis on Randomized Controlled Trials
Source: Rev Cardiovasc Med. 2023 Jan 12;24(1):24. doi: 10.31083/j.rcm2401024 (PMC11270471; doi:10.31083/j.rcm2401024)
Supplement: Supplementary file 1 [file 2153-8174-24-1-024-s1.zip › Supplementary Tables/Table S3. Detailed definitions of outcomes analyzed in current study.docx]

**Table S3**. Detailed definitions of outcomes analyzed in current study

**MACE**:

Defined as cardiovascular death, myocardial infarction (MI), ischaemic stroke and systemic embolism

**MI**:

An event was considered to be an MI if there was evidence of cardiac necrosis (consistent elevation in cardiac biomarkers or relevant autopsy findings) and there was other evidence of an acute MI (including symptoms of ischemia, recent coronary intervention, death, new ECG changes, evidence of a new myocardial defect on cardiac imaging or an acute coronary occlusion at angiography) and no other diagnosis was likely. Troponin was the preferred cardiac biomarker but creatine kinase (CK) or CK-MB was acceptable. The interpretation of the cardiac biomarker depended on the clinical setting but, except in the context of coronary revascularization, at least one value above the upper reference limit was considered to indicative of MI. If original ECG tracings or cardiac biomarker results were not available, it was acceptable to rely upon a report of the findings, e.g., in a discharge summary. Silent MI is not included.

**CHD**:

New Angina: New onset of typical angina with documented ischemia by stress testing (ECG, ECHO or nuclear) in a patient without angina at baseline. Clarification: New angina also includes the following two types of cases: • Angina occurring 6 months or more after the CABG/PCI • Angina occurring any time after CABG/PCI if the subject did not have angina before the procedure

Worsening Angina: Known angina increasing in frequency, duration, and/or severity, and requiring increased anti-anginal medication or coronary revascularization procedure.

Unstable Angina: Unstable angina is defined as ischemic symptoms: (pain, dyspnea, pressure) at rest or accelerated ischemic symptoms, either of which lasts ≥ 10 minutes, that the investigator determines is secondary to ischemia and requires hospitalization.

Silent MI: It is acknowledged that there are instances where myocardial necrosis attributed to MI occurs, and which is clinically unrecognized. If the investigator (based on review of the clinical status and ECGs) feels that this occurred, he/she should submit information supporting the diagnosis of a clinically unrecognized MI. Support would require an ECG showing new and significant Q-waves not attributed to intraventricular conduction defect, left ventricular hypertrophy, pre-excitation syndrome, or electronic pacer. In addition, confirmation may be achieved by echocardiography or other evidence of new regional wall motion abnormalities. The Event Adjudication Committee (EAC) will evaluate clinically reported events in a blinded fashion and ascertain whether they have sufficient information to concur that a significant event, which was clinically unrecognized, has occurred. The timing of that event would be the earliest ECG showing new Q-waves. The EAC may request additional ECG tracings.

Non-fatal MI and fatal MI: specially, fatal MI includes Death occurring within 30 days of a documented MI in which there is no conclusive evidence of another cause of death. Patients who are being treated for MI and who have a sudden death as the terminal event related to the MI will be classified as having a MI-related death. • Autopsy evidence of a recent MI with no other conclusive evidence of another cause of death. • A fatal MI may be adjudicated for death that has suggestive criteria for an MI but does not meet the strict definition of a MI. The suggestive criteria are presentation of chest pain and any one of the following: - ECG changes indicative of a myocardial injury (including new left bundle branch block) or - Abnormal cardiac markers below the level of diagnostic myocardial necrosis: CKMB greater than 1.0 but less than 1.5 X ULN or Troponin or other markers above normal range but less than twice the value of necrosis (e.g., in such an event patient died before a subsequent draw) or Other evidence of new wall motion abnormality.

**Revascularisation:**

Mainly regarding the coronary revascularization and non-coronary revascularization. Coronary revascularization included coronary angioplasty, stenting or coronary artery bypass grafting. Non-coronary revascularization included peripheral angioplasty or stenting, atherectomy, thrombectomy, embolectomy, catheter directed thrombolysis, arterial bypass surgery and aneurysm repair (surgical or endovascular). Arterial embolization (e.g. cranial aneurysm coiling or embolization procedures to treat hemorrhage), amputation procedures and procedures on the venous or pulmonary systems were not included. Attempted procedures which were not completed because of technical difficulties were considered to be a revascularization procedure.

**Stroke**:

Stroke was defined as an acute symptomatic episode of focal or global neurological dysfunction caused by brain, spinal or retinal vascular injury as a result of hemorrhage or infarction which lasts >24 hours, lead to death or was associated with evidence of an acute infarct or hemorrhage on brain imaging corresponding with the clinical syndrome. Strokes were further subdivided by etiology, including confirmed ischemic, confirmed hemorrhagic or uncertain etiology. Hemorrhagic conversion of an ischemic/embolic infarct was considered to be an ischemic stroke. Confirmed hemorrhagic stroke included focal or global neurological dysfunction caused by subarachnoid or intracerebral hemorrhages in the absence of a secondary cause such as trauma.

**Sudden Cardiac Death**:

(1) Death that is witnessed occurring suddenly and unexpectedly in the absence of any non cardiovascular cause. (2) Death that is witnessed occurring within 60 minutes of symptoms onset with no evidence of any non-cardiovascular cause. (3) Death occurring during resuscitation attempts after cardiac arrest or within one month from resuscitation, with no evidence of any other non-cardiovascular cause.

**CV mortality**:

Defined as a death for which a definite non-cardiovascular cause (e.g. cancer) has not been identified. Uncertain causes of deaths are presumed to be cardiovascular. Therefore, cardiovascular deaths include deaths from sudden unexpected death, non-sudden arrhythmic death, un-witnessed death, fatal myocardial infraction, heart failure death, death after invasive cardiovascular intervention, death due to stroke, other cardiovascular death, presumed cardiovascular Death, death from unknown cause.

**All-cause mortality**:

Death from cardiovascular cause and non-cardiovascular cause.

**Hospitalization**:

Ordinary hospital admission with all reasons. Emergency admission was also considered.

**Hospitalization for all heart disease**:

All ordinary hospital admissions are coded by the Coordinating Center using ICD9. Cardiovascular hospitalizations are defined as any admission coded by the Coordinating Center by a term in the ICD9 that maps to the circulatory system (390-459) or interventions on the cardiovascular system (35-39).

**Hospitalization for heart failure**:

Hospitalization for heart failure (new or recurrent) is defined as overnight (two calendar days) hospitalization or attendance in an acute care setting for two of the three following criteria: 1) either signs or symptoms of heart failure and/or 2) radiologic evidence of congestive heart failure and/or 3) requiring intravenous or a first dose or increased dose of oral diuretic, intravenous or oral vasodilator and/or intravenous or oral inotrope.
