# Supplementary material for: Omega-3 Polyunsaturated Fatty Acids Supplements and Cardiovascular Disease Outcome: A Systematic Review and Meta-Analysis on Randomized Controlled Trials
Source: Rev Cardiovasc Med. 2023 Jan 12;24(1):24. doi: 10.31083/j.rcm2401024 (PMC11270471; doi:10.31083/j.rcm2401024)
Supplement: Supplementary file 1 [file 2153-8174-24-1-024-s1.zip › Supplementary Tables/Table S4. Methodology quality of the included studies.docx]

**Table S4**. Methodology quality of the included studies

|  | Random sequence generation *(Selection bias)* | Allocation concealment *(Selection bias)*‡ | Blinding of participants and personnel *(Performance bias)* | Blinding of outcome assessment *(Detection bias)* | Incomplete outcome data *(Attrition bias)* | Selective reporting *(Reporting bias)* | Other sources of bias^§^ |
| --- | --- | --- | --- | --- | --- | --- | --- |
| Marchioli et al^6^, 1999 | Low risk of bias | Low risk of bias | Low risk of bias | Low risk of bias | Low risk of bias | Low risk of bias | unclear^§^ |
| Nilsen et al^7^, 2001 | High risk of bias | High risk of bias | High risk of bias^*^ | High risk of bias^*^ | unclear^§^ | Low risk of bias | Low risk of bias |
| Brouwer et al^8^, 2006 | Low risk of bias | Low risk of bias | Low risk of bias | Low risk of bias | High risk of bias | Low risk of bias | Low risk of bias |
| Svensson et al^9^, 2006 | Low risk of bias | Low risk of bias | Low risk of bias | Low risk of bias | Low risk of bias | Low risk of bias | unclear^§^ |
| Yokoyama et al^10^, 2007 | Low risk of bias | Low risk of bias | High risk of bias^*^ | Low risk of bias | Low risk of bias | Low risk of bias | Low risk of bias |
| Tavazzi et al^11^, 2008 | Low risk of bias | Low risk of bias | Low risk of bias | Low risk of bias | Low risk of bias | Low risk of bias | Low risk of bias |
| Einvik et al^12^, 2010 | Low risk of bias | High risk of bias | High risk of bias^*^ | High risk of bias^*^ | unclear^§^ | Low risk of bias | unclear^§^ |
| Galan et al^13^, 2010 | Low risk of bias | Low risk of bias | Low risk of bias | Low risk of bias | Low risk of bias | Low risk of bias | Low risk of bias |
| Kromhout et al^14^, 2010 | Low risk of bias | Low risk of bias | Low risk of bias | Low risk of bias | High risk of bias | Low risk of bias | Low risk of bias |
| Rauch et al^15^, 2010 | Low risk of bias | Low risk of bias | Low risk of bias | Low risk of bias | Low risk of bias | Low risk of bias | Low risk of bias |
| Bosch et al^16^, 2012 | Low risk of bias | Low risk of bias | Low risk of bias | Low risk of bias | Low risk of bias | Low risk of bias | Low risk of bias |
| Macchia et al^17^, 2013 | Low risk of bias | Low risk of bias | Low risk of bias | Low risk of bias | Low risk of bias | Low risk of bias | unclear^§^ |
| Roncaglioni et al^18^, 2013 | Low risk of bias | Low risk of bias | Low risk of bias | Low risk of bias | Low risk of bias | Low risk of bias | Low risk of bias |
| Bonds et al^19^, 2014 | Low risk of bias | Low risk of bias | Low risk of bias | Low risk of bias | High risk of bias | Low risk of bias | unclear^§^ |
| Nosaka et al^20^, 2017 | Low risk of bias | Low risk of bias | Low risk of bias | High risk of bias^*^ | unclear^§^ | Low risk of bias | unclear^§^ |
| Bowman et al^21^, 2018 | Low risk of bias | Low risk of bias | High risk of bias† | High risk of bias† | Low risk of bias | Low risk of bias | Low risk of bias |
| Bhatt et al^22^, 2019 | Low risk of bias | Low risk of bias | Low risk of bias | Low risk of bias | Low risk of bias | Low risk of bias | Low risk of bias |
| Nicholls et al^23^, 2020 | Low risk of bias | Low risk of bias | Low risk of bias | Low risk of bias | Low risk of bias | Low risk of bias | Low risk of bias |
| Kalstad et al^24^, 2021 | Low risk of bias | Low risk of bias | Low risk of bias | Low risk of bias | Low risk of bias | Low risk of bias | Low risk of bias |

^*^There is no clear description on the blinding methods.

†Open label randomized controlled trials or no blinding methods performed.

‡Allocation concealment was missed in open-label trial

^§^Can not give precise judgements on other quality-assessment items.
