# Supplementary figures and images for: Omega-3 Polyunsaturated Fatty Acids Supplements and Cardiovascular Disease Outcome: A Systematic Review and Meta-Analysis on Randomized Controlled Trials
Source: Rev Cardiovasc Med. 2023 Jan 12;24(1):24. doi: 10.31083/j.rcm2401024 (PMC11270471; doi:10.31083/j.rcm2401024)

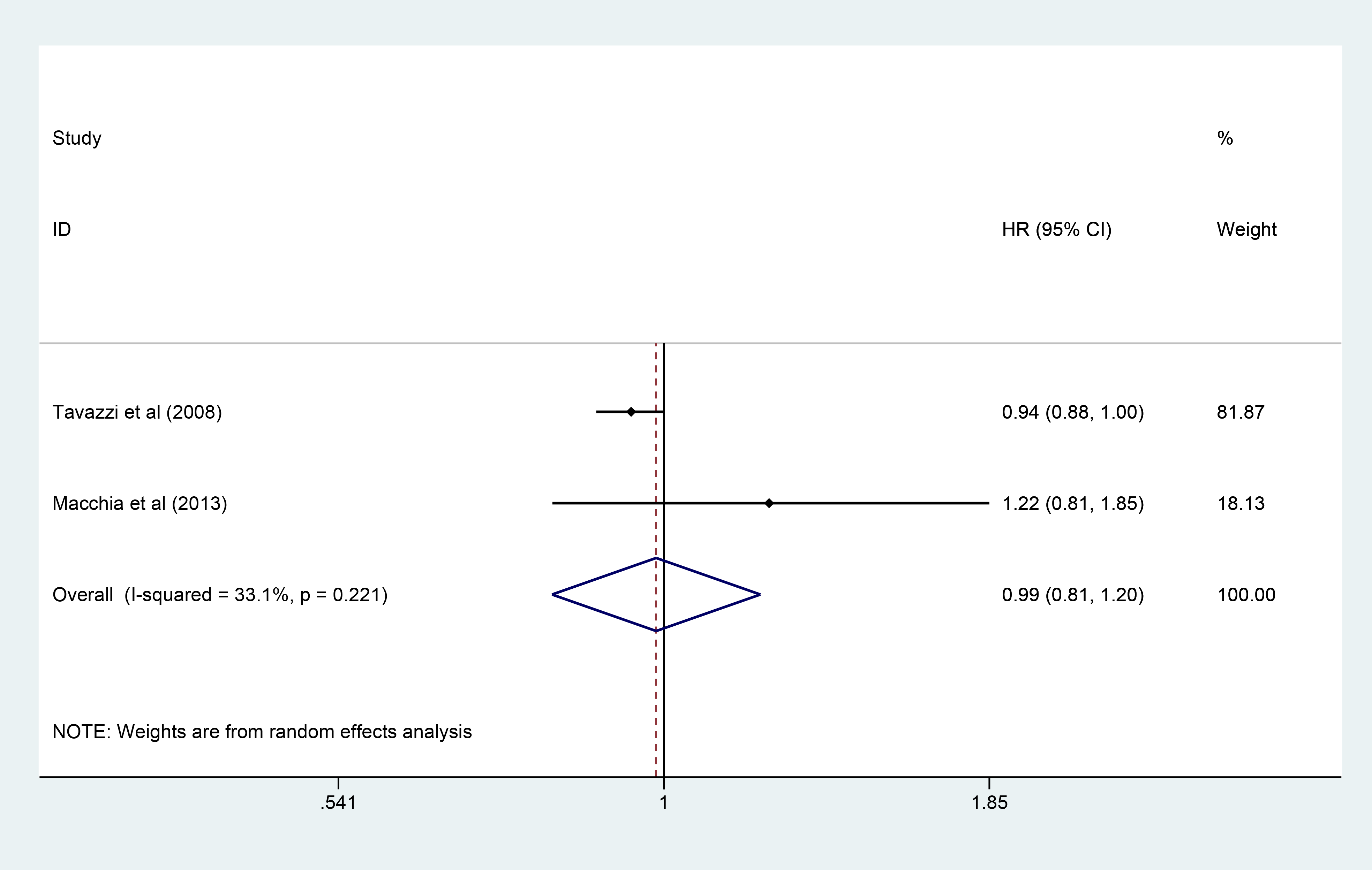

Supplement: Supplementary file 1 [file 2153-8174-24-1-024-s1.zip › Supplementary Figures/Figure S1.tif]

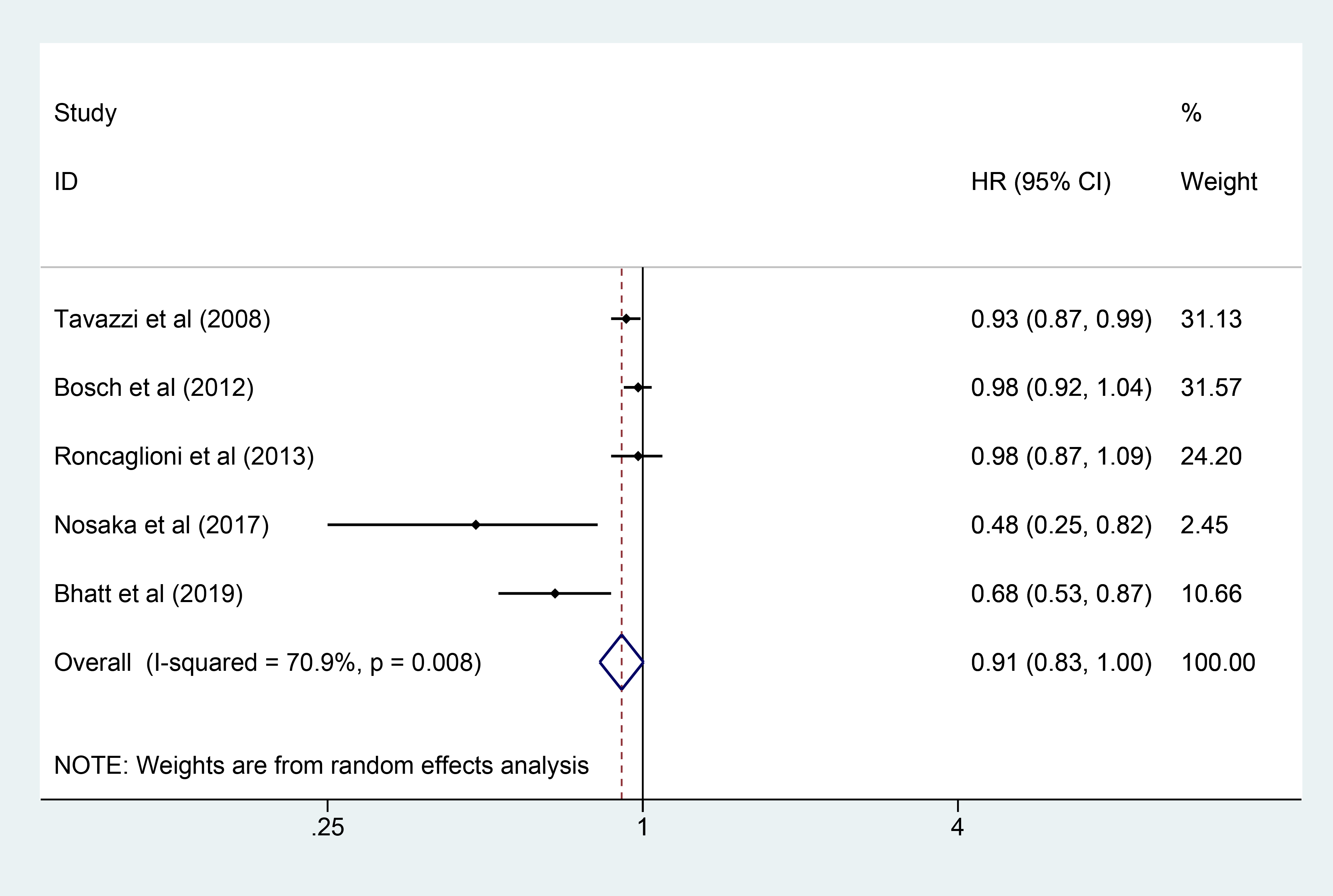

Supplement: Supplementary file 1 [file 2153-8174-24-1-024-s1.zip › Supplementary Figures/Figure S2.tif]

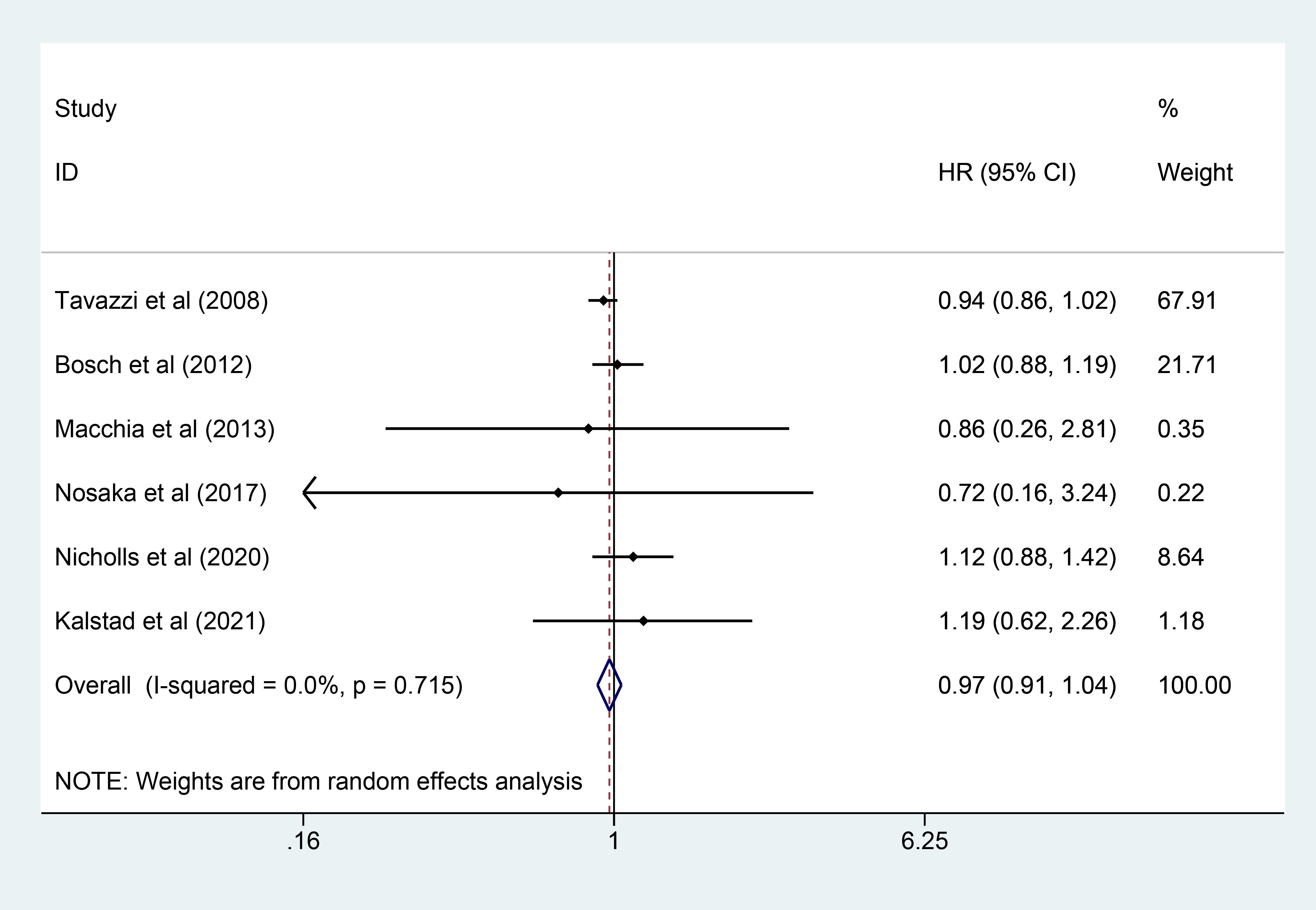

Supplement: Supplementary file 1 [file 2153-8174-24-1-024-s1.zip › Supplementary Figures/Figure S3.tif]

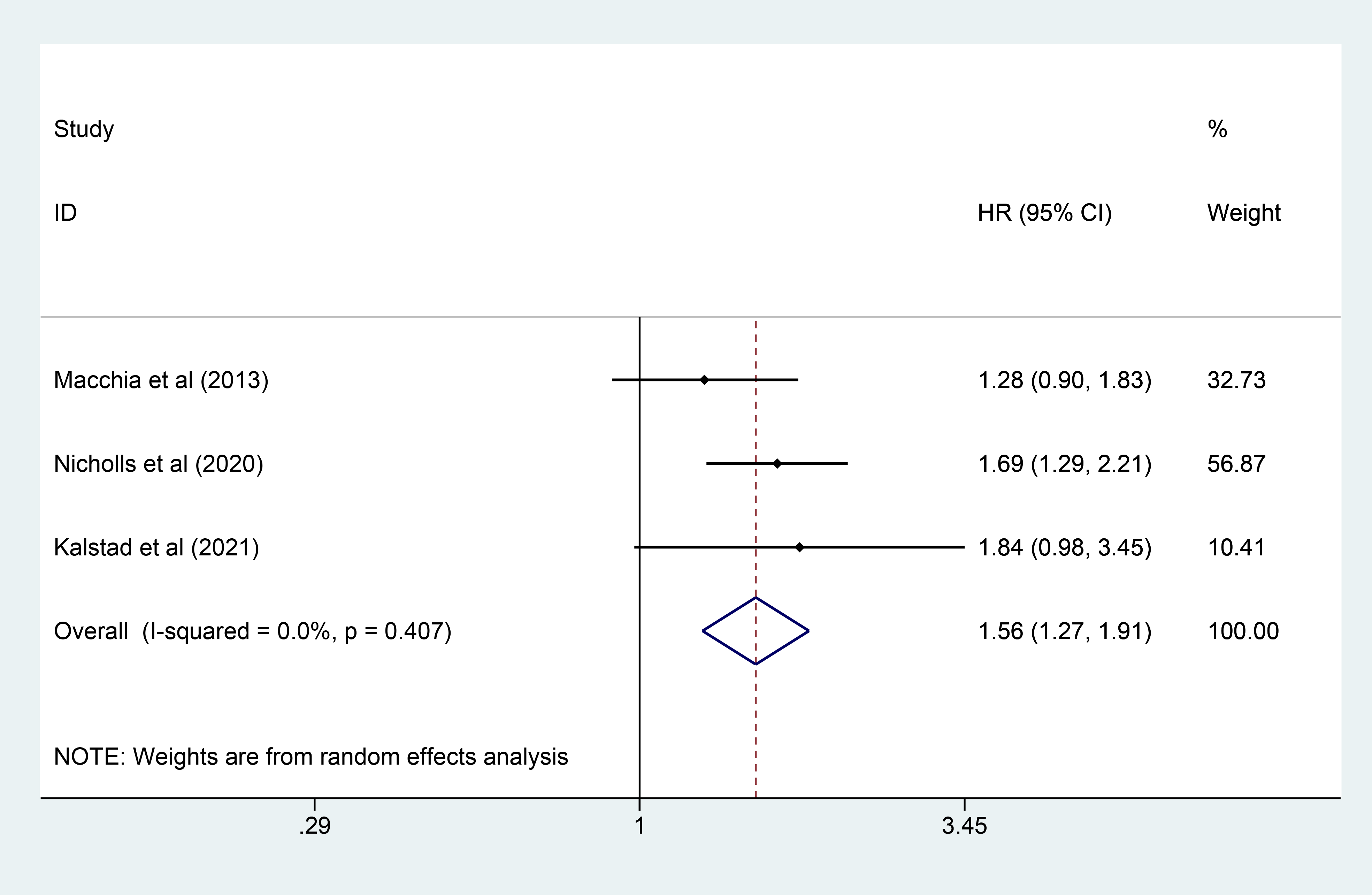

Supplement: Supplementary file 1 [file 2153-8174-24-1-024-s1.zip › Supplementary Figures/Figure S4.tif]
